# IscR Is Essential for *Yersinia pseudotuberculosis* Type III Secretion and Virulence

**DOI:** 10.1371/journal.ppat.1004194

**Published:** 2014-06-12

**Authors:** Halie K. Miller, Laura Kwuan, Leah Schwiesow, David L. Bernick, Erin Mettert, Hector A. Ramirez, James M. Ragle, Patricia P. Chan, Patricia J. Kiley, Todd M. Lowe, Victoria Auerbuch

**Affiliations:** 1 Department of Microbiology and Environmental Toxicology, University of California Santa Cruz, Santa Cruz, California, United States of America; 2 Biomolecular Engineering, University of California Santa Cruz, Santa Cruz, California, United States of America; 3 Department of Biomolecular Chemistry, University of Wisconsin-Madison, Madison, Wisconsin, United States of America; 4 Department of Molecular, Cell, and Developmental Biology, University of California Santa Cruz, Santa Cruz, California, United States of America; University of California San Diego, United States of America

## Abstract

Type III secretion systems (T3SS) are essential for virulence in dozens of pathogens, but are not required for growth outside the host. Therefore, the T3SS of many bacterial species are under tight regulatory control. To increase our understanding of the molecular mechanisms behind T3SS regulation, we performed a transposon screen to identify genes important for T3SS function in the food-borne pathogen *Yersinia pseudotuberculosis*. We identified two unique transposon insertions in YPTB2860, a gene that displays 79% identity with the *E. coli* iron-sulfur cluster regulator, IscR. A *Y. pseudotuberculosis iscR* in-frame deletion mutant (Δ*iscR*) was deficient in secretion of Ysc T3SS effector proteins and in targeting macrophages through the T3SS. To determine the mechanism behind IscR control of the Ysc T3SS, we carried out transcriptome and bioinformatic analysis to identify *Y. pseudotuberculosis* genes regulated by IscR. We discovered a putative IscR binding motif upstream of the *Y. pseudotuberculosis yscW-lcrF* operon. As LcrF controls transcription of a number of critical T3SS genes in *Yersinia*, we hypothesized that *Yersinia* IscR may control the Ysc T3SS through LcrF. Indeed, purified IscR bound to the identified *yscW-lcrF* promoter motif and mRNA levels of *lcrF* and 24 other T3SS genes were reduced in *Y. pseudotuberculosis* in the absence of IscR. Importantly, mice orally infected with the *Y. pseudotuberculosis* Δ*iscR* mutant displayed decreased bacterial burden in Peyer's patches, mesenteric lymph nodes, spleens, and livers, indicating an essential role for IscR in *Y. pseudotuberculosis* virulence. This study presents the first characterization of *Yersinia* IscR and provides evidence that IscR is critical for virulence and type III secretion through direct regulation of the T3SS master regulator, LcrF.

## Author Summary

Bacterial pathogens use regulators that sense environmental cues to enhance their fitness. Here, we identify a transcriptional regulator in the human gut pathogen, *Yersinia pseudotuberculosis*, which controls a specialized secretion system essential for bacterial growth in mammalian tissues. This regulator was shown in other bacterial species to alter its activity in response to changes in iron concentration and oxidative stress, but has never been studied in *Yersinia*. Importantly, *Y. pseudotuberculosis* experiences large changes in iron bioavailability upon transit from the gut to deeper tissues and iron is a critical component in *Yersinia* virulence, as individuals with iron overload disorders have enhanced susceptibility to systemic *Yersinia* infections. Our work places this iron-modulated transcriptional regulator within the regulatory network that controls virulence gene expression in *Y. pseudotuberculosis*, identifying it as a potential new target for antimicrobial agents.

## Introduction

Type III secretion systems (T3SS) are important components in the progression of disease for a number of clinically relevant human pathogens, including those in the genera *Shigella*, *Salmonella*, *Escherichia*, *Chlamydia*, *Vibrio*, *Pseudomonas*, and *Yersinia* [1], [2]. The T3SS functions as an injectisome that delivers bacterial effector proteins directly into the host cell cytoplasm [2]. While the T3SS apparatus itself is structurally conserved, the repertoire of T3SS effector proteins used by each group of pathogens is distinct [2]. Thus, the effect of the T3SS on the host is unique to the needs of the pathogen [2]. While the T3SS is generally essential for a T3SS-expressing pathogen to cause disease, several aspects of the T3SS may be detrimental to bacterial growth [2]. For example, T3SS components are recognized by the host immune system [3], [4]. In addition, expression of the T3SS is energetically costly and, in some organisms, T3SS induction correlates with growth arrest [5]. Therefore, regulation is essential for proper T3SS function in order to ensure that it occurs only during host cell contact in the appropriate host tissue [2], [6].

Members of the genus *Yersinia* that utilize a T3SS are important human pathogens: *Y. pestis*, the causative agent of plague, and the enteropathogens *Y. enterocolitica* and *Y. pseudotuberculosis*. The *Y. pseudotuberculosis* Ysc T3SS is encoded on a 70-kb plasmid termed pYV [7]–[9] and is made up of approximately 25 known proteins comprising three main structures: the basal body, the needle apparatus, and the translocon [10], [11]. The basal body, which displays a high degree of similarity to the flagellar basal body, is made up of rings that span the inner and outer membranes and a rod that traverses the periplasmic space [12]. Basal body associated proteins include YscN, an ATPase that aids in the secretion and translocation of effector proteins [13]. The needle complex, which is thought to act as a molecular channel for effector protein translocation, is a straight hollow appendage approximately 60 nm in length and is made up of helical polymerized subunits of YscF [12]. The translocon is comprised of three proteins: YopD, YopB and LcrV, which are essential for pore formation in the target host membrane and proper translocation of effector proteins YopHEMOJTK to the host cytoplasm [12], [14]. Also encoded on pYV are chaperones important for efficient translocation of a subset of effector proteins [15]. Lastly, several transcriptional and post-transcriptional regulators of the T3SS are found on pYV, including the AraC-like transcriptional regulator LcrF. LcrF is responsible for expression of a number of T3SS structural genes and Yop effectors, specifically the *virC* and *lcrGVH-yopBD* operons as well as genes encoding effector Yops, the adhesin YadA, and the lipoprotein YlpA [16]-[22]. LcrF itself is thermoregulated at both the transcriptional and translational levels through the action of the histone-like protein YmoA and a cis-acting RNA thermosensor located on the *lcrF* transcript, respectively [23], [24]. This enables *Yersinia* to express T3SS genes at 37°C within the mammalian host, but not at lower temperatures [23], [24]. Importantly, proper LcrF-mediated control of T3SS expression is important for *Y. pseudotuberculosis* virulence [24].

IscR belongs to the Rrf2 family of winged helix-turn-helix transcription factors [25], [26] and has been studied extensively in *E. coli*, where its DNA-binding activity is dependent on coordination of an iron-sulfur [2Fe-2S] cluster through three conserved cysteines and a histidine [27]–[30]. *E. coli* IscR recognizes two distinct DNA motifs, type 1 and type 2, depending on the Fe-S status of the protein [31]. Holo-IscR coordinating an Fe-S cluster binds both type 1 and type 2 motifs, while clusterless apo-IscR recognizes only the type 2 DNA-binding motif [27], [32], [33]. As iron starvation, oxidative stress, and oxygen limitation affect the holo-IscR/apo-IscR ratio, these environmental cues are thought to have a direct effect on gene expression through IscR in *E. coli* [28]–[30]. For example, holo-IscR represses transcription of the housekeeping *iscRSUA*-*hscBA*-*fdx* Fe-S cluster biogenesis operon [32], [34], while either holo- or apo-IscR promotes transcription of the inducible *sufABCDSE* Fe-S cluster biogenesis operon [33], [35]. Both pathways function to insert Fe-S clusters onto proteins involved in a range of metabolic processes including electron transfer, substrate binding/activation, iron/sulfur storage, regulation, and enzyme activity [36]. In addition, *E. coli* IscR is also known to regulate transcription of other Fe-S cluster assembly genes such as *erpA* (*yadR*) as well as genes integral to oxidative stress resistance, biofilm formation, and anaerobic respiration [28]–[30], [34]. IscR is widely conserved among bacteria [25] and its regulatory activity is integral to the infectious process of the plant pathogen *Erwinia chrysanthemi* [37]. Furthermore, IscR plays an important role in the virulence of the human pathogens *Pseudomonas aeruginosa* through modulation of the catalase *katA* [38], *Burkholderia mallei* through resistance to reactive nitrogen species [39], and *Vibrio vulnificus* through induction of several virulence-associated pathways [39], [40]. While the iron-dependent transcriptional repressor Fur has been shown to control T3SS expression in *Salmonella* and *Shigella* [41], [42], IscR has never been linked to regulation of the T3SS in any organism and has not been studied in *Yersinia*.

In this study, we isolated two independent IscR transposon insertion mutants in a novel screen for *Y. pseudotuberculosis* genes important for T3SS function. We assessed the impact of *iscR* deletion on *Y. pseudotuberculosis in vitro* and *in vivo* growth, type III secretion, and global gene expression. We found IscR to be essential for full T3SS function and virulence in a mouse model of infection. In addition, we provide evidence that IscR control of the T3SS stems from direct transcriptional regulation of the T3SS master regulator LcrF.

## Results

### IscR is required for *Y. pseudotuberculosis* Ysc T3SS function

**Figure 1:**
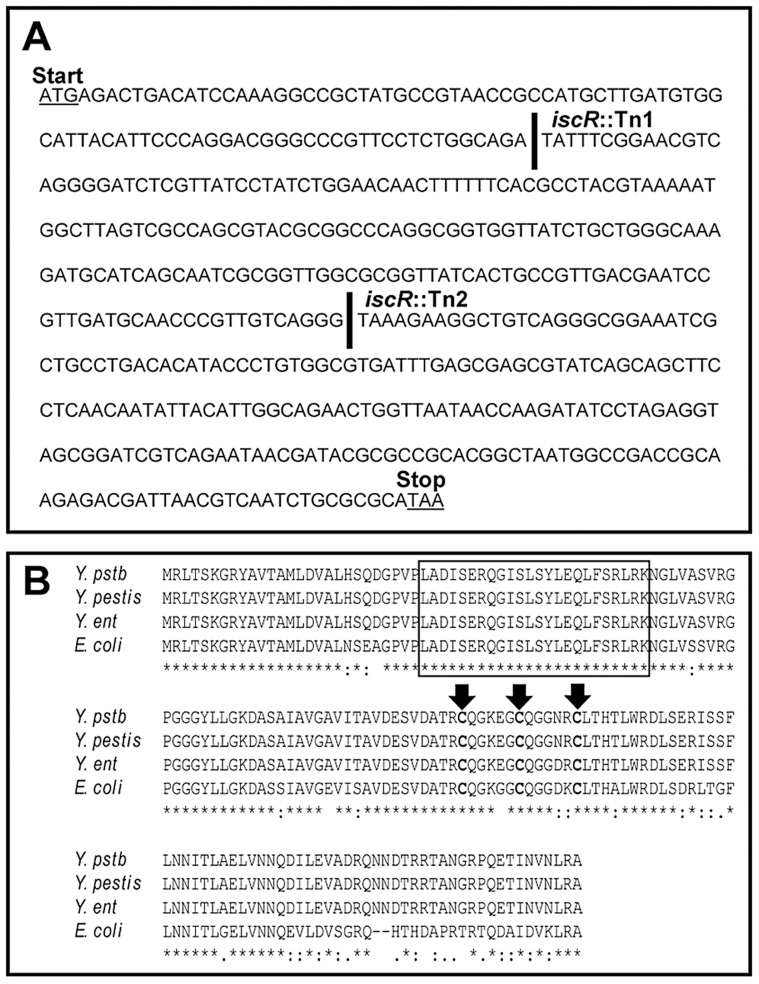
Alignment of protein sequences shows a high level of conservation between *E. coli* and *Yersinia* IscR. (**A**) The *Y. pseudotuberculosis* DNA sequence, which displays the unique insertions sites for the two transposon mutants generated from our genetic screen. A space in the DNA sequence and a solid black line indicate the site of insertion for either *iscR*::Tn1 or *iscR*::Tn2. (**B**) Multiple sequence alignment was performed on the IscR protein sequence from *E. coli* K12-MG1655 and each of the three human pathogenic *Yersinia* spp., *Y. pseudotuberculosis* IP 32953 (*Y. pstb*), *Y. enterocolitica* 8081 (*Y. ent*) and *Y. pestis* CO92 (*Y. pestis*) using ClustalW [86]. The N-terminal helix-turn-helix DNA-binding motif is indicated by a black box. The three conserved cysteine residues (C92, C98 and C104) responsible for coordinating an Fe-S cluster are in bold and identified by black arrows [33]. Asterisks indicate nucleotides that are conserved across all four species.

To identify regulators of the *Y. pseudotuberculosis* T3SS, we utilized a novel screen to isolate transposon mutants with defects in T3SS function. We previously showed that *Y. pseudotuberculosis* expressing a functional T3SS induces NFκB activation in HEK293T cells [43], enabling us to use host cell NFκB activation as a readout for T3SS function in *Y. pseudotuberculosis* transposon mutants. As some T3SS effector proteins inhibit NFκB signaling [44], we performed the screen using a *Y. pseudotuberculosis* transposon mutant library in a genetic background that lacked the known T3SS effector proteins YopHEMOJT (Δyop6; [43]). We identified several transposon mutants with defects in triggering activation of NFκB in HEK293T cells (L. Kwuan, N. Herrera, H. Ramirez, V. Auerbuch, data not shown), suggesting defective T3SS function. Among these were two strains with unique transposon insertions in YPTB2860 (Figure 1A), encoding a protein with 79% identity to the *E. coli* iron-sulfur cluster regulator IscR, part of the *iscRSUA-hscBA-fdx* operon involved in Fe-S cluster biogenesis (Figure 1B). Importantly, the helix-turn-helix DNA binding domain as well as the three cysteines and histidine known to coordinate an iron-sulfur (Fe-S) cluster in *E. coli* IscR are conserved in all three *Yersinia* species (Figure 1B). These data indicate that *Yersinia* IscR may coordinate an Fe-S cluster and, as in *E. coli*, may regulate gene transcription.

**Figure 2:**
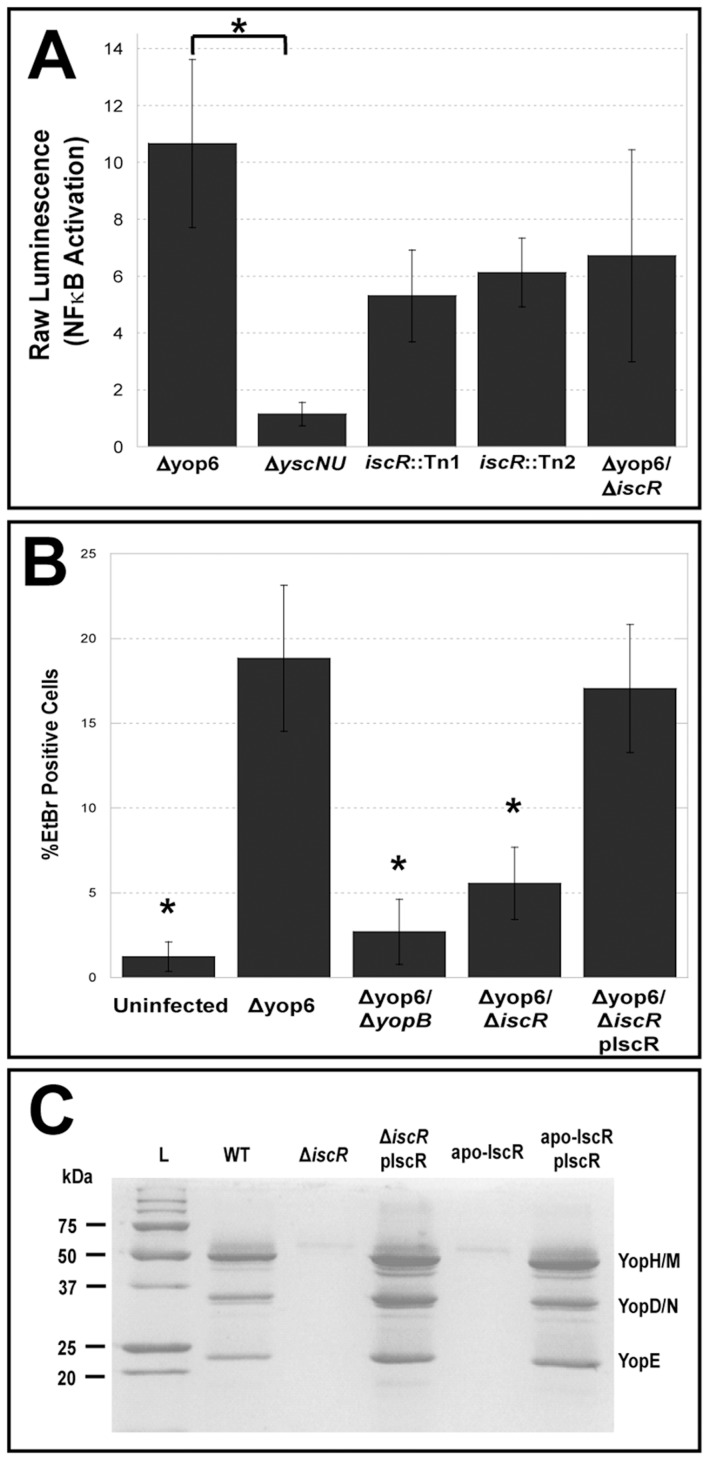
IscR modulates *Y. pseudotuberculosis* T3SS function. (**A**) HEK293T cells expressing an NFκB luciferase reporter gene were infected with either T3SS-positive *Y. pseudotuberculosis* lacking the six known effector proteins YopHEMOJT (Δyop6), two isogenic *iscR* transposon mutants (Δyop6/*iscR*::Tn1 and Δyop6/*iscR*::Tn2), the *iscR* deletion mutant (Δyop6/Δ*iscR*), or a T3SS null mutant (Δ*yscNU*). At 4 h post-inoculation, T3SS functionality was determined by assessing the ability of the mutants to trigger NFκB activation in host cells by measuring bioluminescence. Shown are the average raw luminescence values of the sample compared to uninfected ± standard error of the mean (SEM) from five independent experiments. *p≤0.05, as determined by one-way ANOVA followed by Bonferroni post hoc test where each indicated group was compared to the appropriate negative (Δ*yscNU*) and positive (Δyop6) controls. (**B**) To analyze T3SS-dependent pore formation in macrophages, C57Bl/6 immortalized BMDMs were infected with Δyop6, a T3SS-defective mutant lacking the translocator protein YopB (Δyop6/Δ*yopB*), the *iscR* deletion mutant (Δyop6/*iscR*), or the *iscR* complemented strain (Δyop6/*iscR* pIscR), or were left uninfected. At 2 h post-inoculation, pore formation was determined by assessing the number of cells that took up ethidium bromide (EtBr) compared to the total number of Hoechst-stained cells. Shown are the averages ± SEM from three independent experiments. *p≤0.05 relative to both Δyop6 and Δyop6/*iscR* pIscR, as determined by one-way ANOVA followed by Bonferroni post hoc test where each indicated group was compared to the appropriate negative (Δyop6/Δ*yopB*) and positive (Δyop6) controls. (**C**) *Y. pseudotuberculosis* IP2666 wild type (WT), *iscR* deletion (Δ*iscR*), *iscR* complemented (Δ*iscR* pIscR), apo-locked IscR (apo-IscR), and apo-IscR complemented (apo-IscR pIscR) strains were grown in 2xYT low calcium media at 37°C to induce type III secretion in the absence of host cells. Proteins in the bacterial culture supernatant were precipitated and visualized alongside a protein molecular weight marker (L) on a polyacrylamide gel using commassie blue. Sample loading was normalized for OD_600_ of each culture. Results are representative of three independent experiments.

To validate that loss of IscR in *Y. pseudotuberculosis* leads to T3SS defects, we isolated the two *iscR* transposon mutants (*iscR*::Tn1 and *iscR*::Tn2) from our library and again measured their ability to trigger NFκB activation in HEK293T cells compared to the Δyop6 parental strain and a Δ*yscNU* T3SS-null mutant [43]. In addition, we constructed an in-frame *iscR* deletion mutant in the Δyop6 genetic background (Δyop6/Δ*iscR*) and tested it in this assay. We found that disruption of *iscR* led to ∼2-fold less NFκB activation relative to the Δyop6 T3SS^+^ parental strain, although NFκB activation levels were still ∼5-fold higher than a strain with complete lack of T3SS function (Δ*yscNU*; Figure 2A), suggesting that loss of *iscR* leads to partial T3SS loss.

To verify further that deletion of *iscR* leads to alterations in T3SS function, we assessed the ability of the Δyop6/Δ*iscR* mutant to insert YopBD pores in target host cell membranes by measuring entry of ethidium bromide (EtBr) inside *Y. pseudotuberculosis*-infected bone marrow derived macrophages [45], [46]. Pore formation by the Δyop6/Δ*iscR* mutant was decreased by 7-fold (p<0.05) relative to the Δyop6 parental strain, which could be restored upon complementation with plasmid-encoded *iscR* (Figure 2B). To determine whether loss of *iscR* affects T3SS function in a wild type genetic background, we constructed an in-frame *iscR* deletion (Δ*iscR*) in the wild type *Y. pseudotuberculosis* IP2666 strain expressing six of the seven known T3SS effector proteins YopHEMOJK [47]. We then visualized the secretome of the Δ*iscR* mutant relative to wild type. Deletion of *iscR* led to a dramatic decrease in secretion of T3SS cargo relative to the wild type background, which can be restored upon complementation with plasmid-encoded *iscR* (Figure 2C). Importantly, this lack of type III secretion did not result from a defect in growth of the mutant, as the Δ*iscR* mutant actually grew better than wild type bacteria under T3SS-inducing conditions (Figure S1A). This is consistent with a T3SS defect in this strain, as wild type *Yersinia* display a characteristic growth arrest upon T3SS expression [5], [48], [49]. Collectively, these data demonstrate that *Y. pseudotuberculosis* IscR is required for proper T3SS function.

### IscR is required for full virulence of *Y. pseudotuberculosis*

**Figure 3:**
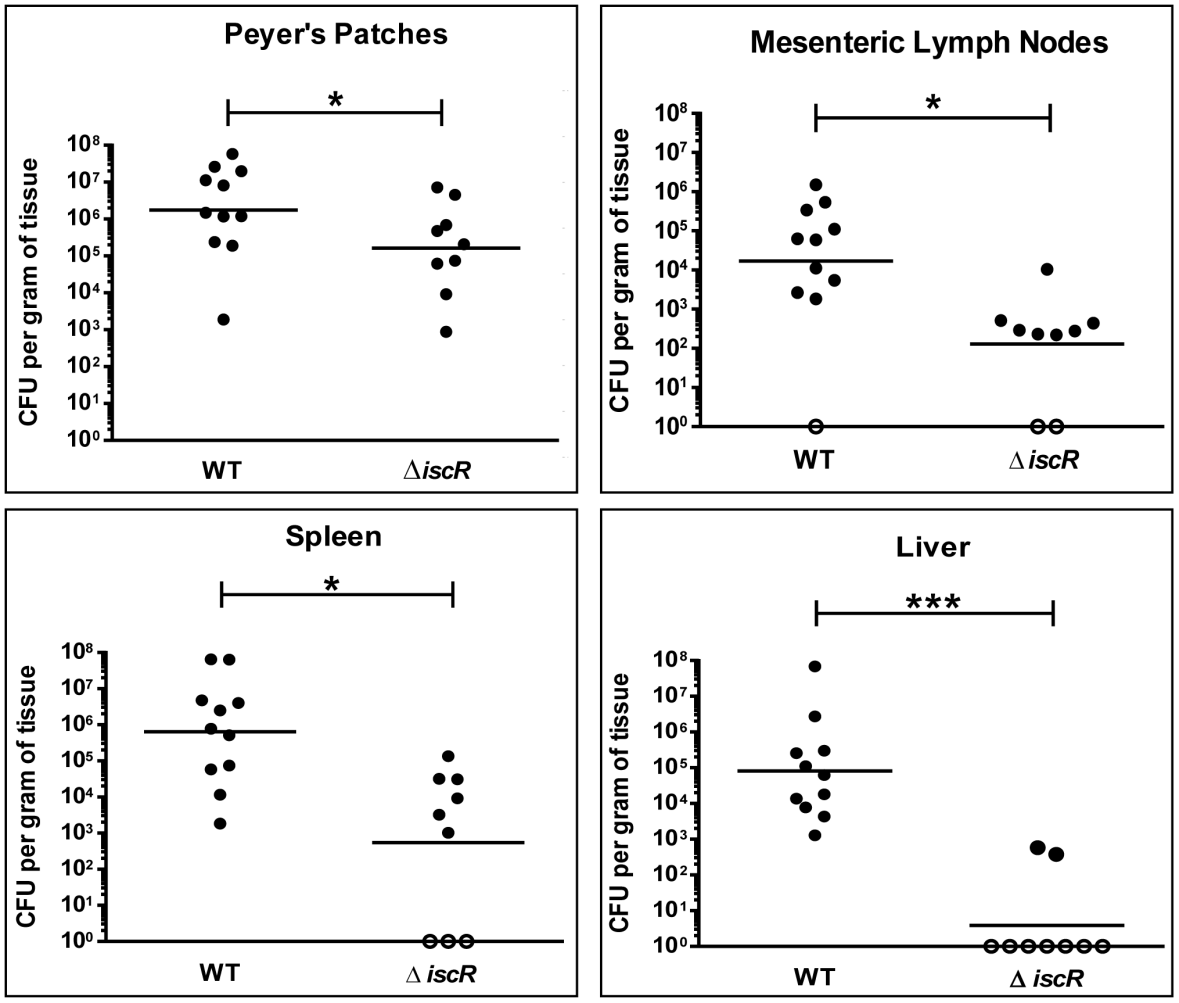
IscR is required for full virulence of *Y. pseudotuberculosis*. Mice were infected with 2×10^8^ CFU of either WT *Y. pseudotuberculosis* or Δ*iscR* mutant via orogastric gavage. At 5 days post-inoculation, the Peyer's patches (PP), mesenteric lymph nodes (MLN), spleens and livers were collected, homogenized and CFU determined. Each symbol represents one animal. Unfilled symbols indicate that CFU were below the limit of detection. The data presented are from three independent experiments. *p<0.05, ***p<0.001 as determined by an unpaired Wilcoxon-Mann-Whitney rank sum test. Dashes represent the geometric mean.

Based on the knowledge that the T3SS plays an important role in the virulence of human pathogenic *Yersinia*, we sought to investigate whether the diminished type III secretion observed in the *Y. pseudotuberculosis* Δ*iscR* strain would lead to a reduction in the infectious capacity of this mutant. Mice were orogastrically infected with 2×10^8^ CFU of either the *Y. pseudotuberculosis* wild type or isogenic Δ*iscR* mutant strains. At 5 days post-inoculation, mice infected with the Δ*iscR* mutant displayed significantly decreased colonization of Peyer's patches and mesenteric lymph nodes (MLN) as well as diminished systemic colonization (Figure 3). Specifically, we noted 10- and 130-fold reductions in CFU recovered from the Peyer's patches and MLNs, respectively, in mice infected with the Δ*iscR* mutant strain relative to wild type. Notably, we observed a 1000- to 10,000-fold decrease in bacterial burden in the spleen and liver respectively. The diminished ability of the Δ*iscR* mutant strain to colonize deep tissue sites is underscored by the fact that bacteria were not detected in seven of the nine livers analyzed. These findings suggest that IscR is essential for *Y. pseudotuberculosis* virulence in an oral infection model.

### IscR deletion leads to global misregulation of gene expression in *Y. pseudotuberculosis*

To begin to understand the mechanistic contribution of IscR to *Y. pseudotuberculosis* pathogenesis, we performed high throughput transcriptome sequencing (RNAseq) analysis to determine the *Y. pseudotuberculosis* genes directly and indirectly controlled by IscR under iron replete, T3SS-inducing conditions. Total RNA was collected from wild type *Y. pseudotuberculosis* as well as the Δ*iscR* mutant strain grown in M9 at 37°C for 3 h, a point at which the Δ*iscR* and wild type strains display comparable growth rates (Figure S1A).

**Figure 4:**
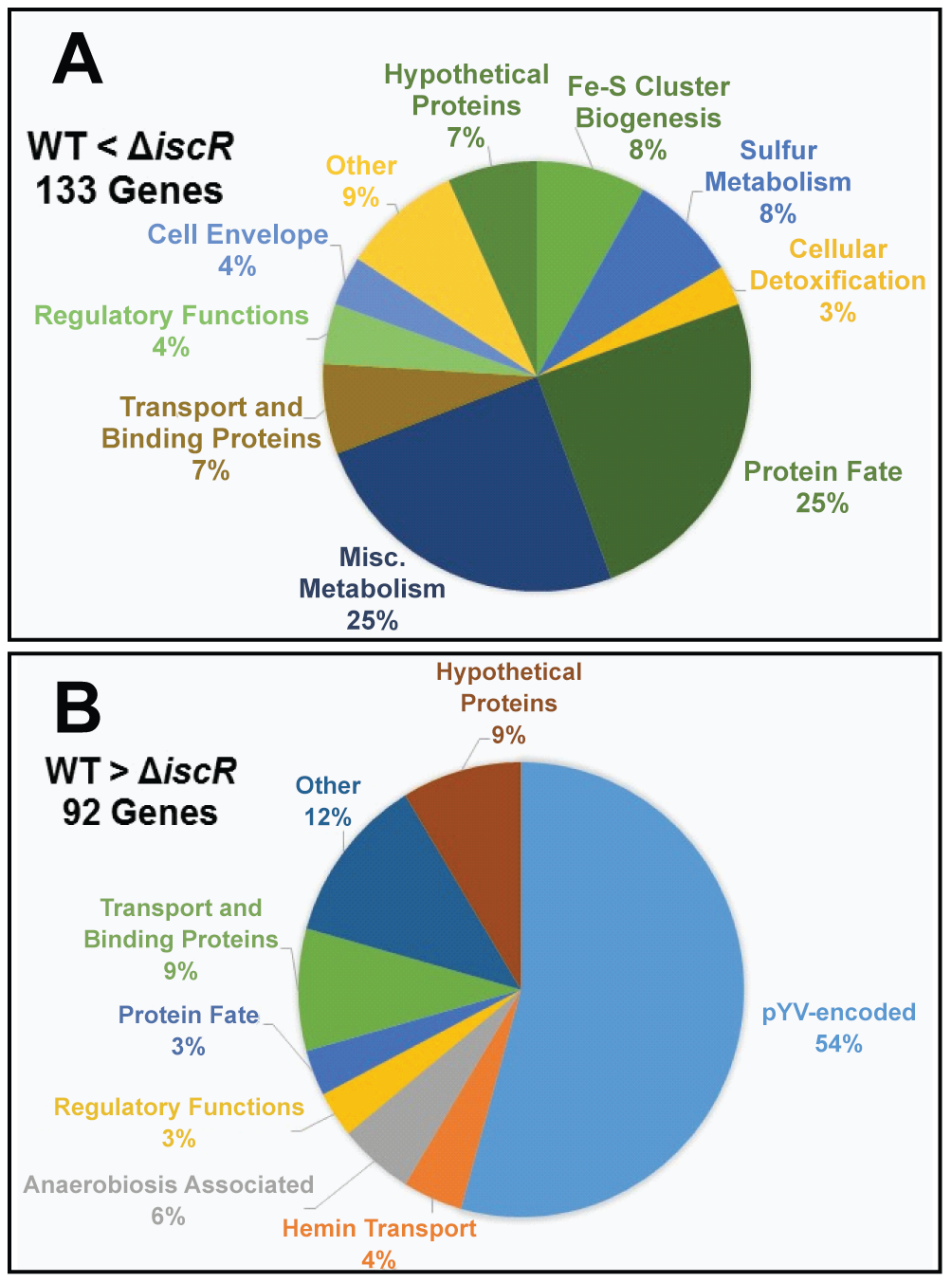
IscR impacts global gene expression in *Y. pseudotuberculosis* under iron replete conditions. RNAseq analysis was performed on WT and Δ*iscR Y. pseudotuberculosis* after growth in M9 at 37°C for 3 h (T3SS-inducing conditions), at which point total RNA was collected and processed. The resulting libraries were sequenced using the HiSeq2500 Illumina sequencing platform for 50 bp single reads and analyzed via the CLC Genomics Workbench application (CLC bio). RPKM expression levels of 225 genes demonstrated a fold change of ≥2, and were deemed significant by Bayseq test with a corrected FDR post hoc test from three independent experiments (p≤0.05). Shown are the functional ontologies of the (**A**) 133 genes that are up-regulated in the Δ*iscR* mutant relative to the wild type and (**B**) 92 that are down-regulated.

**Figure 5:**
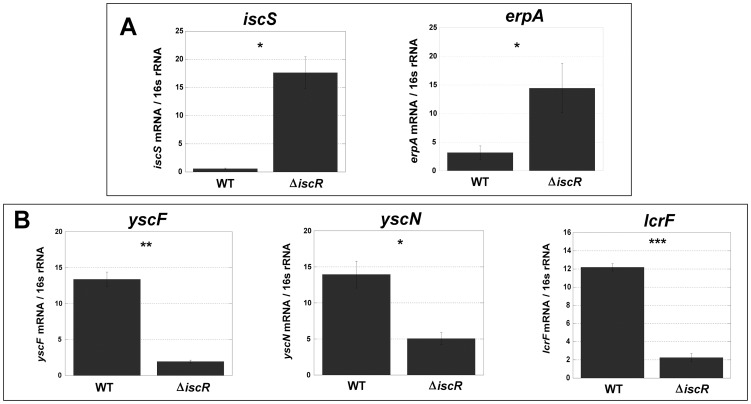
Deletion of IscR leads to increased transcription of Fe-S cluster biogenesis genes and robust transcription of T3SS genes. Quantitative real-time PCR analysis of WT and Δ*iscR Y. pseudotuberculosis* was performed (A) for the Fe-S cluster biogenesis genes, *iscS* and *erpA* and (B) for the T3SS genes, *yscF*, *yscN* and *lcrF*. Experiments were carried out from cultures grown in M9 at 37°C for 3 h. Shown are the averages ± SEM from three independent experiments. *p<0.05, **p<0.001, ***p<0.0001 as determined by a Student *t* test.

For the Δ*iscR* mutant relative to the wild type, a total of 226 genes demonstrated a statistically significant fold change of ≥2 (Table S1). Of these, 134 genes were up-regulated in the Δ*iscR* mutant relative to the wild type (Table 1 & Figure 4A), while 92 were down-regulated (Table 2 & Figure 4B). Genes found to be up-regulated in the Δ*iscR* mutant include key elements of Fe-S cluster biosynthesis, cellular detoxification, metabolism, and protein fate (Figure 4A). The most notable increases in transcription were observed for genes encoding Fe-S cluster biosynthesis proteins including those encoded in the *isc* operon, *iscS* (18.7-fold), *iscU* (21.7-fold) and *iscA* (13-fold) (Table 1 & Figure S2A). Additional genes encoding proteins involved in Fe-S cluster assembly and their respective fold increases include *iscX/yfhJ* (10.8), *fdx* (10.9), *hscB* (10), *hscA* (9.3), *yadR/erpA* (6.8), *pepB* (10.1) and *nfuA* (7.0). To validate these findings, we performed qRT-PCR analysis on the second gene encoded in the *iscRSUA* operon, *iscS*, as well as on the gene encoding the Fe-S biosynthesis protein ErpA. Transcription of *iscS* was increased by 30-fold, while *erpA* expression was increased 5-fold (p<0.05; Figure 5A). Bioinformatic analysis identified two IscR type 1 motifs upstream of the *iscRSUA-hscBA-fdx* operon (Figure S2B) as well as one site each located upstream of both *erpA* and *nfuA* (data not shown). Based on this data, we propose that *Y. pseudotuberculosis* IscR modulates Fe-S cluster biosynthesis expression in a manner akin to that of *E. coli* IscR.

**Table 1:** Genes repressed by IscR, identified by RNAseq analysis.

Gene Ontology	ORF ID^a^	Description	Gene			Fold Up Regulation^b^
Fe-S Cluster Biogenesis (11)	YPTB0744	Fe-S insertion protein	*yadR/erpA*			6.8
	YPTB2851	enhanced serine sensitivity protein	*sseB*			6.6
	YPTB2852	peptidase B	*pepB*			10.1
	YPTB2853	Fe-S assembly protein	*iscX/yfhJ*			10.8
	YPTB2854	Isc system ferredoxin	*fdx*			10.9
	YPTB2855	Fe-S assembly chaperone	*hscA*			9.3
	YPTB2856	Fe-S assembly chaperone	*hscB*			10.0
	YPTB2857	Fe-S assembly protein	*iscA*			13.0
	YPTB2858	Fe-S assembly scaffold	*iscU*			21.7
	YPTB2859	cysteine desulfurase	*iscS*			18.7
	YPTB3773	Fe-S biogenesis protein	*nfuA*			7.0
Sulfur Metabolism (11)	YPTB0759	sulfite reductase, beta (flavoprotein) subunit	*cysJ*			3.7
	YPTB0760	sulfite reductase, alpha subunit	*cysI*			2.2
	YPTB0761	3-phosphoadenosine 5-phosphosulfate (PAPS) reductase	*cysH*			2.0
	YPTB0764	Siroheme synthase	*cysG*			2.4
	YPTB0765	ATP-sulfurylase, subunit 2	*cysD*			4.8
	YPTB0766	ATP-sulfurylase, subunit 1	*cysN*			4.0
	YPTB0767	adenosine 5-phosphosulfate kinase	*cysC*			2.7
	YPTB2714	cysteine synthase A	*cysK*			3.5
	YPTB2732	ABC sulfate transporter, ATP-binding subunit	*cysA*			2.1
	YPTB2735	ABC trans, periplasmic thiosulfate-binding protein	*cysP*			3.0
	YPTB2769	putative sulfatase	*ydeN*			2.5
Cellular Detox (4)	YPTB0756	superoxide dismutase precursor (Cu-Zn)	*sodC*			2.0
	YPTB0811	catalase hydroperoxidase HPI(I)	*katY*			8.6
	YPTB2261	thiol peroxidase	*tpx*			2.0
	YPTB2299	superoxide dismutase [Fe]	*sodB*			6.3
Protein Fate (33)	YPTB0017	secreted thiol:disulfide interchange protein	*dsbA*			2.0
	YPTB0097	ATP-binding heat shock protein	*hslU*			2.7
	YPTB0102	50S ribosomal protein L31	*rpmE*			2.4
	YPTB0276	elongation factor Tu	*tuf*			2.7
	YPTB0279	50S ribosomal protein L11	*rplK*			2.5
	YPTB0280	50S ribosomal protein L1	*rplA*			2.3
	YPTB0281	50S ribosomal protein L10	*rplJ*			2.7
	YPTB0282	50S ribosomal protein L7/L12	*rplL*			3.0
	YPTB0404	10 kDa chaperonin	*groES*			5.2
	YPTB0405	60 kDa chaperonin	*groEL*			5.7
	YPTB0438	30S ribosomal protein S6	*rpsF*			2.2
	YPTB0440	30S ribosomal protein S18	*rpsR*			2.2
	YPTB0441	50S ribosomal protein L9	*rplI*			2.3
	YPTB0464	50S ribosomal protein L21	*rplU*			2.3
	YPTB0465	50S ribosomal protein L27	*rpmA*			2.6
	YPTB0611	chaperone Hsp70	*dnaK*			3.8
	YPTB0612	heat shock protein	*dnaJ*			3.6
	YPTB0749	periplasmic serine protease Do, heat shock protein	*htrA*			4.4
	YPTB0848	ATP-dependent, Hsp 100	*clpB*			4.1
Protein Fate (33)	YPTB0958	trigger factor	*tig*			2.5
	YPTB0960	clpA-clpP ATP-dependent serine protease, chaperone	*clpX*			2.7
	YPTB0961	ATP-dependent protease	*lon*			2.9
	YPTB0995	chaperone Hsp90, heat shock protein C 62.5	*htpG*			3.3
	YPTB1090	sec-independent protein translocase protein	*tatE*			2.9
	YPTB1113	putative tRNA-thiotransferase	*miaB*			2.0
	YPTB1141	heat shock protein GrpE	*grpE*			2.7
	YPTB1417	30S ribosomal protein S1	*rpsA*			2.2
	YPTB1448	putative ribosome modulation factor	*rmf*			3.8
	YPTB2820	putative protease				2.1
	YPTB3000	ribosome recycling factor	*frr*			2.1
	YPTB3026	protease III precursor	*ptrA*			2.4
	YPTB3511	Protease	*degQ*			3.4
	YPTB3904	heat shock protein	*ibpA*			3.5
	YPTB3905	heat shock protein	*ibpB*			4.5
Misc. Metabolism (33)	YPTB0135	acetolactate synthase isozyme II small subunit	*ilvM*			2.6
	YPTB0297	DNA-binding protein HU-alpha	*hupA*			2.1
	YPTB0402	aspartate ammonia-lyase	*aspA*			3.1
	YPTB0456	fructose-1, 6-bisphosphatase	*fbp*			2.1
	YPTB0460	malate dehydrogenase	*mdh*			2.1
	YPTB0546	putative glycoprotein/receptor				2.0
	YPTB0755	enolase	*eno*			2.1
	YPTB0809	probable cytochrome b(561)	*cybB*			4.0
	YPTB0810	putative cytochrome b(562)	*cybC*			6.7
	YPTB1117	putative N-acetylglucosamine regulatory protein	*nagC*			2.5
	YPTB1118	N-acetylglucosamine-6-phosphate deacetylase	*nagA*			4.1
	YPTB1119	putative glucosamine-6-phosphate isomerase	*nagB*			5.9
	YPTB1120	N-acetylglucosamine-specific IIABC component	*nagE*			5.2
	YPTB1148	dihydrolipoamide succinyltransferase	*sucB*			2.1
	YPTB1149	succinyl-CoA synthetase beta chain	*sucC*			3.0
	YPTB1150	succinyl-CoA synthetase alpha chain	*sucD*			3.1
	YPTB1358	glutaredoxin 1	*grxA*			2.3
	YPTB1418	integration host factor beta-subunit	*ihfB*			2.7
	YPTB2047	pyruvate kinase II	*pykA*			2.3
	YPTB2143	aconitate hydratase 1	*acnA*			2.2
	YPTB2216	putative acetolactate synthase large subunit	*ilvB*			2.2
	YPTB2217	putative acetolactate synthase small subunit	*ilvN*			2.3
	YPTB2306	pyruvate kinase I	*pykF*			2.0
	YPTB2845	nucleoside diphosphate kinase	*ndk*			2.3
	YPTB2870	flavohemoprotein	*hmp*			2.1
	YPTB2943	urease beta subunit	*ureB*			2.0
	YPTB2944	urease gamma subunit	*ureA*			2.1
	YPTB3202	Biosynthetic arginine decarboxylase	*speA*			2.1
	YPTB3572	biotin carboxylase	*accC*			2.1
	YPTB3966	ATP synthase epsilon subunit protein	*atpC*			2.5
	YPTB3967	ATP synthase beta subunit protein	*atpD*			2.3
	YPTB3968	ATP synthase gamma subunit protein	*atpG*			2.2
	YPTB3969	ATP synthase alpha subunit protein	*atpA*			2.1
Regulatory Functions (6)	YPTB0784	putative transcriptional regulatory protein				2.0
	YPTB1955	putative phosphate starvation-inducible protein	*phoH*			2.2
	YPTB3068	putative carbonic anhydrase				2.2
	YPTB3418	RNA polymerase sigma factor RpoD	*rpoD*			2.3
	YPTB3527	putative sigma N modulation factor	*yhbH*			2.1
Transport and Binding Proteins (9)	YPTB0306	putative sodium:phenylacetate symporter	*actP*			2.4
	YPTB1718	putative cystine-binding periplasmic protein	*fliY*			2.4
	YPTB2463	PTS system, glucose-specific IIBC component	*ptsG*			2.0
	YPTB2682	ABC transporter, periplasmic iron(III)-binding protein	*sfuA*			2.6
	YPTB2717	PTS system glucose-specific IIA component, permease		*crr*		2.3
	YPTB2770	probable ABC transporter, ATP-binding subunit				2.8
	YPTB2771	putative ABC iron transporter				2.7
	YPTB2772	ABC transporter, periplasmmic iron binding protein				4.1
	YPTB3957	ABC transporter, periplasmic amino acid binding protein				4.6
Cell Envelope (5)	YPTB1334	pH 6 antigen precursor (antigen 4) (adhesin)	*psaA*			2.5
	YPTB2123	putative exported protein	*ompW*			3.3
	YPTB2287	putative lipoprotein	*slyB*			2.0
	YPTB2867	attachment invasion locus protein	*ail*			4.3
	YPTB3584	outermembrane protein	*pcp*			2.5
Other (12)	YPTB0439	primosomal replication protein n	*priB*			2.2
	YPTB0693	tubulin-like GTP-binding protein and GTPase	*ftsZ*			2.1
	YPTB0782	putative dihydroxyacetone kinase				2.7
	YPTB0830	quorum sensing protein	*luxS*			2.4
	YPTB1162	quinolinate synthetase A	*nadA*			2.1
	YPTB1182	biotin synthase	*bioB*			2.2
	YPTB1468	cytotoxic necrotizing factor (partial)				6.1
	YPTB1517	formaldehyde dehydrogenase				2.2
	YPTB2248	D-lactate dehydrogenase	*ldhA*			2.1
	YPTB2395	probable N-acetylmuramoyl-L-alanine amidase				2.4
	YPTB2791	putative arsenate reductase	*yfgD*			2.0
	YPTB2887	pyridoxal phosphate biosynthetic protein	*pdxJ*			2.2
				
Hypothetical Proteins(9)	YPTB0391	putative exported protein				2.1
	YPTB0449	hypothetical protein				3.3
	YPTB0458	putative exported protein				2.2
	YPTB1093	hypothetical protein				3.6
	YPTB1571	hypothetical protein				2.1
	YPTB2255	putative exported protein				2.3
	YPTB2277	hypothetical protein				2.6
	YPTB2496	hypothetical protein				2.8
	YPTB3109	hypothetical protein				4.1

a ORF IDs are derived from the *Y. pseudotuberculosis* IP 32593 genome unless otherwise stated.

b Fold change is of the Δ*iscR* mutant relative to the wild type strain.

**Table 2:** Genes activated by IscR, identified by RNAseq analysis.

Gene Ontology	ORF ID^a^	Description	Gene		Fold Up Regulation^b^
pYV-encoded (50)	pYV0002	YpkA chaperone	*sycO*		4.6
	pYV0003	putative transposase remnant			2.2
	pYV0008	possible transposase remnant			2.6
	pYV0009	hyothetical protein			3.3
	pYV0010	hypothetical protein			3.3
	pYV0012	hypothetical protein			4.2
	pYV0014	possible transposase remnant			3.2
	pYV0015	possible transposase remnant			3.9
	pYV0016	tnpA putative transposase protein			2.6
	pYV0021	putative transposase			2.4
	pYV0022	putative transposase			2.3
	pYV0023	possible transposase remnant			2.8
	pYV0034	putative transposase remnant			2.3
	pYV0035	hypothetical protein			3.0
	pYV0036	hypothetical protein			3.6
	pYV0037	C-term conjugative transfer: surface exclusion			4.2
	pYV0038	N-term fragment conjugative transfer: surface exclusion			8.3
	pYV0039	putative transposase			7.5
	pYV0040	yop targeting protein	*yopK*		9.3
	pYV0041	yop targeted effector	*yopT*		5.5
	pYV0044	hypothetical protein			4.1
	pYV0046	putative transposase remnant			2.9
	pYV0047	targeted effector protein	*yopM*		5.3
	pYV0049	hypothetical protein			2.4
	pYV0056	low calcium response protein H	*lcrH*		3.9
	pYV0057	V antigen, antihost protein/regulator	*lcrV*		3.5
	pYV0058	Yop regulator	*lcrG*		2.8
	pYV0061	type III secretion protein	*yscY*		2.2
	pYV0062	type III secretion protein	*yscX*		2.5
	pYV0063	type III secretion protein	*sycN*		2.5
	pYV0064	Yop secretion and targeting protein	*tyeA*		2.1
	pYV0068	type III secretion protein	*yscO*		2.0
	pYV0069	type III secretion protein	*yscP*		2.1
	pYV0075	Yop targeting lipoprotein	*virG*		2.5
	pYV0076	putative thermoregulatory protein	*lcrF*		3.3
	pYV0078	hypothetical protein	*yscB*		3.5
	pYV0079	type III secretion protein	*yscC*		2.0
	pYV0080	type III secretion protein	*yscD*		2.0
	pYV0082	type III secretion protein	*yscF*		2.8
	pYV0083	type III secretion protein	*yscG*		2.9
	pYV0084	type III secretion protein	*yscH*		2.1
	pYV0087	type III secretion protein	*yscK*		3.2
	pYV0088	type III secretion protein	*yscL*		2.2
	pYV0089	type III secretion regulatory	*lcrQ*		2.1
	pYV0090	putative transposase			2.7
	pYV0091	putative transposase			3.1
	pYV0092	putative transposase			3.2
	pYV0093	putative transposase			2.2
	pYV0098	targeted effector protein	*yopJ*		3.4
	pYV0099	hypothetical protein			4.8
Hemin Transport (4)	YPTB0336	ABC hemin transporter, ATP-binding subunit	*hmuV*		2.4
	YPTB0337	ABC hemin transporter, permease subunit	*hmuU*		2.4
	YPTB0338	ABC transporter, periplasmic hemin-binding protein	*hmuT*		2.4
	YPTB0339	hemin degradation/transport protein	*hmuS*		2.2
Anaerobiosis Associated (5)	YPTB0209	anaerobic glycerol-3-phosphate dehydrogenase subunit A	*glpA*		2.3
	YPTB0518	anaerobic ribonucleotide reductase activating protein	*nrdG*		2.6
	YPTB0805	anaerobic dimethyl sulfoxide reductase, subunit A	*dmsA*		2.3
	YPTB0806	anaerobic dimethyl sulfoxide reductase, subunit B	*dmsB*		2.1
	YPTB2688	putative dimethyl sulfoxide reductase chain A protein	*dmsA*		2.1
Regulatory Functions (3)	YPTB0247	lysR-family transcriptional regulatory protein	*metR*		2.0
	YPTB0386	L-rhamnose operon regulatory protein	*rhaS*		2.4
	YPTB3808	putative hybrid two-component system regulatory protein			2.0
Protein Fate (3)	YPTB0495	putative protease			3.0
	YPTB0877	translation initiation factor EIF-2B, GDP-GTP exchange factor (alpha subunit)	*eif*		2.2
	YPTB1266	putative outer membrane-associated protease	*pla2*		2.2
Transport and Binding Proteins (8)	YPTB0502	ABC type sugar transport system, permease			2.0
	YPTB0868	putative amino acid ABC transporter, permease			2.5
	YPTB1724	SSS family proline symporter	*putP*		2.4
	YPTB1956	calcium/proton antiporter	*chaA*		2.7
	YPTB2011	SulP family sulfate permease	*ychM*		2.1
	YPTB2022	MFS multidrug efflux antiporter	*yceL*		2.1
	YPTB2491	proton dependent di-tripeptide transporter	*yceE*		2.0
	YPTB2815	AcrB/AcrD/AcrF (HAE1) family drug efflux pump	*yegO*		2.2

a ORF IDs are derived from the *Y. pseudotuberculosis* IP 32593 genome unless otherwise stated.

b Fold change is of the Δ*iscR* mutant relative to the wild type strain.

### IscR is required for transcription of T3SS genes

In total, 92 genes were significantly down-regulated in the Δ*iscR* mutant relative to wild type *Y. pseudotuberculosis* (Table 2). These data demonstrate that the majority of pYV-encoded genes are decreased in the Δ*iscR* mutant relative to the wild type strain, including genes essential for proper T3SS expression and function. The *virC* and *lcrGVH*-*yopBD* operons as well as genes encoding the T3SS cargo YopHEMOJTK were the most affected upon deletion of *iscR*: the effector proteins YopJ (−3.4-fold), YopM (−5.3-fold) and YopT (−5.5-fold), the effector protein and translocation regulator YopK (−9.3-fold), as well as a number of genes encoding T3SS structural proteins. Genes encoding regulators that control T3SS expression and function were decreased in the mutant including *lcrQ* (−2.1-fold), *lcrF* (−3.3-fold), *lcrG* (−2.8-fold) and *lcrH* (−3.9-fold). To verify that T3SS gene expression was indeed decreased in the Δ*iscR* mutant, we measured the transcript levels of the genes encoding T3SS structural proteins YscN, YscF, and the T3SS transcriptional regulator LcrF via qRT-PCR. As detailed in Figure 5B, we observed fold decreases of 2.8-fold (p<0.05), 6.9-fold (p<0.001), and 5.4-fold (p<0.0001) for *yscN*, *yscF,* and *lcrF*, respectively. These data support our RNAseq analysis and confirm that IscR is required for robust transcription of *Y. pseudotuberculosis* T3SS genes.

In addition to T3SS genes, 25 other pYV-encoded genes were decreased in the mutant, but these are annotated as hypothetical proteins, transposases, and pseudogenes. Analysis of the relative abundance of pYV in the *Y. pseudotuberculosis* wild type and Δ*iscR* strains was performed in order to verify that the decreases in pYV-encoded genes were not a result of plasmid loss (Figure S3). The concentration of plasmid isolated from the wild type and Δ*iscR* mutant was comparable, suggesting that the decreased transcription of pYV-encoded genes, including those encoding the T3SS, are not a result of decreased stability of the pYV plasmid.

### *Y. pseudotuberculosis* expressing only apo-locked IscR has a proton motive force defect and cannot secrete Yops

To assess the contribution of Fe-S cluster ligation to IscR control of the T3SS, we constructed an IscR mutant strain in which the three conserved cysteines were substituted with alanines (C92A, C98A, C104A; apo-locked IscR). Identical mutations in *E. coli* IscR render the protein unable to coordinate an iron-sulfur cluster, yet able to bind type 2 DNA binding motifs and to regulate target gene transcription [28]–[30]. We analyzed the secretome of the *Y. pseudotuberculosis* apo-locked IscR strain under T3SS-inducing conditions and found that the mutant was just as defective as the Δ*iscR* strain in Yop secretion (Figure 2C). This defect could be complemented with plasmid-encoded wild type IscR. As apo-locked IscR is insufficient to promote type III secretion, holo-IscR-mediated regulation of gene expression through one or more type 1 motifs may be specifically involved in regulating T3SS gene expression. Alternatively, forcing all IscR expression within the cell to the clusterless form, which leads to IscR overexpression, may lead to alterations of bacterial pathways that indirectly affect type III secretion.

**Figure 6:**
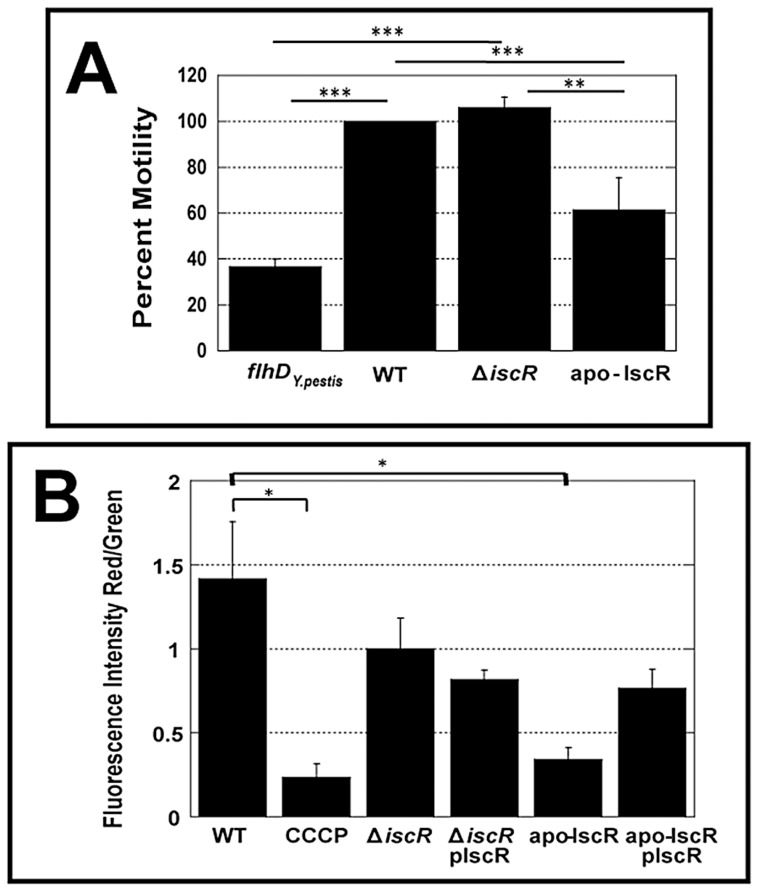
The apo-IscR mutant strain displays decreased motility and disruption of electrical potential. (**A**) Motility was analyzed by spotting 1 µl aliquots of either a nonmotile strain (Δyop6/*flhD^Y.pestis^*), WT, Δ*iscR*, or apo-locked IscR *Y. pseudotuberculosis* onto motility agar plates. The diameters of the colonies were determined one day later and used to calculate percent motility relative to WT, which was set at 100%. Shown is the average percent motility ± SEM and is representative of three independent experiments. ***p≤0.0001 as determined by one-way ANOVA followed by Bonferroni post hoc test where each indicated group was compared to the appropriate negative (Δyop6/*flhD^Y.pestis^*) and positive (WT) controls. (**B**) Proton motive force (PMF) was measured using JC-1 dye for *Y. pseudotuberculosis* IP2666 wild type (WT), *iscR* deletion mutant (Δ*iscR*), *iscR* complemented (Δ*iscR* pIscR), apo-IscR, and apo-IscR complemented (apo-IscR pIscR) strains grown in M9 at 37°C for 3 hours. The protonophore CCCP was added to a WT sample as a negative control (CCCP). Decreases in PMF were measured as a decrease in red (590 nm) fluorescent cells relative to green (530 nm). The data is presented as total fluorescence intensities at 590 (red) relative to 530 (green) ± SEM and is representative of three independent experiments. *p≤0.05, as determined by one-way ANOVA followed by Bonferroni post hoc test where each indicated group was compared to the appropriate negative (CCCP) and positive (WT) controls.

Consistent with this latter explanation, the apo-locked IscR mutant exhibited decreased colony size on LB agar, slower growth in rich media (Figure S1), and decreased motility (Figure 6A). The flagellar basal body is a T3SS itself, indicating that the defect in the Ysc T3SS for this strain may be a result of gross abnormalities in secretion systems. Based on these findings, we set out to examine whether the apo-locked IscR mutant demonstrated alterations in membrane potential, as this has been shown to be important for both motility and Ysc T3S in *Y. enterocolitica* [50]. To this end, we examined bacterial membrane potential under T3SS inducing conditions. As demonstrated in Figure 6B, there is a notable decrease in membrane potential in the apo-locked IscR mutant relative to the wild type strain, which can be complemented upon addition of wild type *iscR* on a plasmid. Furthermore, the membrane potential of the Δ*iscR* mutant strain is comparable to that of the wild type. Collectively, these data suggest that the apo-locked IscR mutant has a proton motive force defect, leading to decreased type III secretion and motility. These findings highlight the importance for *Yersinia* to maintain appropriate levels of holo-IscR relative to apo-IscR in order maintain normal membrane potential.

### IscR recognizes a type 2 motif upstream of the *yscW-lcrF* operon

To begin to understand the nature of the T3SS defect in the presence of only apo-IscR, we carried out RNAseq analysis on the *Y. pseudotuberculosis* apo-locked IscR mutant grown under T3SS-inducing conditions and compared the results with data from the wild type and Δ*iscR* strains. Curiously, the apo-locked IscR mutant displayed aberrant expression of genes involved in stress response, transport, cell envelope, as well as electron transport (data not shown). Of note, the Fe-S cluster biosynthesis proteins encoded in the *iscRSUA-hscBA-fdx-iscX-pepB-sseB*, *yadR/erpA* and *nfuA* operons are significantly increased in this background, similar to that of the Δ*iscR* mutant strain (Figure S2A), indicating that holo-IscR represses expression of these genes under the aerobic, iron-replete conditions used. In contrast, increases in the *sufABCDS* Fe-S cluster biogenesis operon were observed for the apo-locked IscR strain when compared to both the wild type and Δ*iscR* strains (Figure S4). As IscR is overexpressed by 30-fold (p<0.05) in the apo-locked *iscR* mutant compared to wild type (Figure S2A), we speculate that the *suf* operon is positively regulated by IscR in *Yersinia* as in *E. coli*. In contrast, the extensively studied *E. coli* IscR target, *hyaABCDEF*, is not encoded in the *Y. pseudotuberculosis* genome.

**Figure 7:**
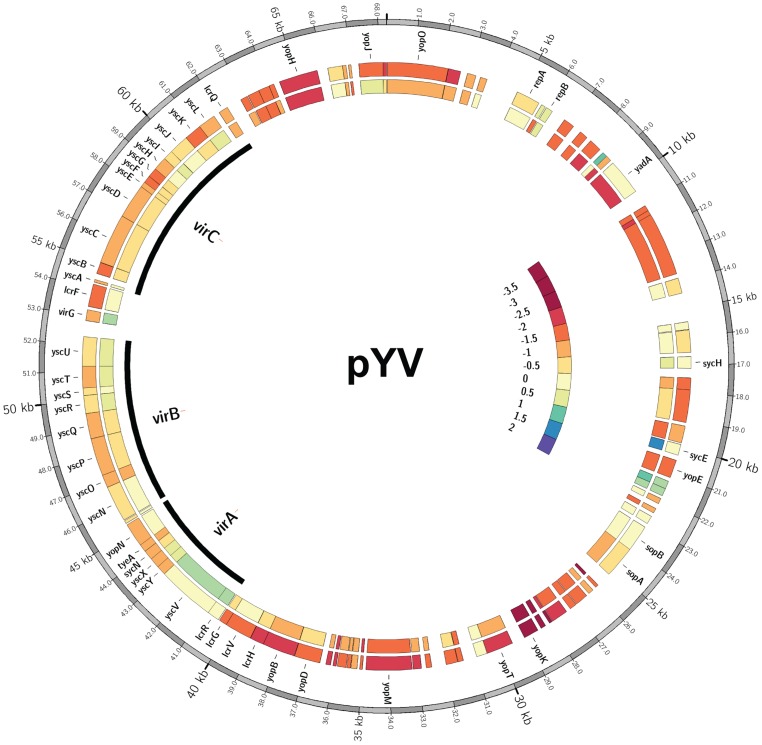
*Y. pseudotuberculosis* lacking a functional IscR display decreased transcription of a number of pYV encoded genes. Middle and inner rings: heatmap [83] representations of log_2_-ratios (log_2_(RPKM_mutant_/RPKM_wt_) for each gene on the pYV plasmid for both the Δ*iscR* (middle ring) and apo-IscR (inner ring) mutants relative to wild type. Outer ring: pYV base coordinate position from *Y. pseudotuberculosis* IP32953. Known genes are identified and the *virA*, *virB* and *virC* operons highlighted by black arcs. On the interior right side is the color bar legend displaying log_2_-ratios from −3.5 to 2. Using this scale, orange/red colorations represent genes with decreased transcription in the mutant relative to the wild type strain and blue/green coloring represents increases in gene transcription for the mutant relative to the wild type. Tan/cream denotes no change.

Importantly, our RNAseq analysis demonstrated that transcription of genes within the *virA*, *virB*, *virC*, *yscW-lcrF*, and *lcrGVH-yopBD* operons was restored in the apo-locked IscR mutant compared to the Δ*iscR* mutant (Figure 7 and Table S2). However, we observed a decrease in transcription of genes encoding the T3SS effector proteins YopH (−4.4-fold), YopM (−3.0-fold), YopK (−7.1-fold), and YopE (−2.1-fold) in the apo-locked IscR mutant compared to wild type. Transcription of *yopE* has been shown to be regulated by Yop secretion through a positive feedback loop [51], [52], suggesting that the defect in YopHEMK transcription observed in the apo-locked IscR mutant may be caused by the lack of Yop secretion we observed in this strain. Together, these data suggest that both holo- and apo-IscR can promote T3SS gene transcription, possibly through binding to one or more type 2 DNA motifs.

**Figure 8:**
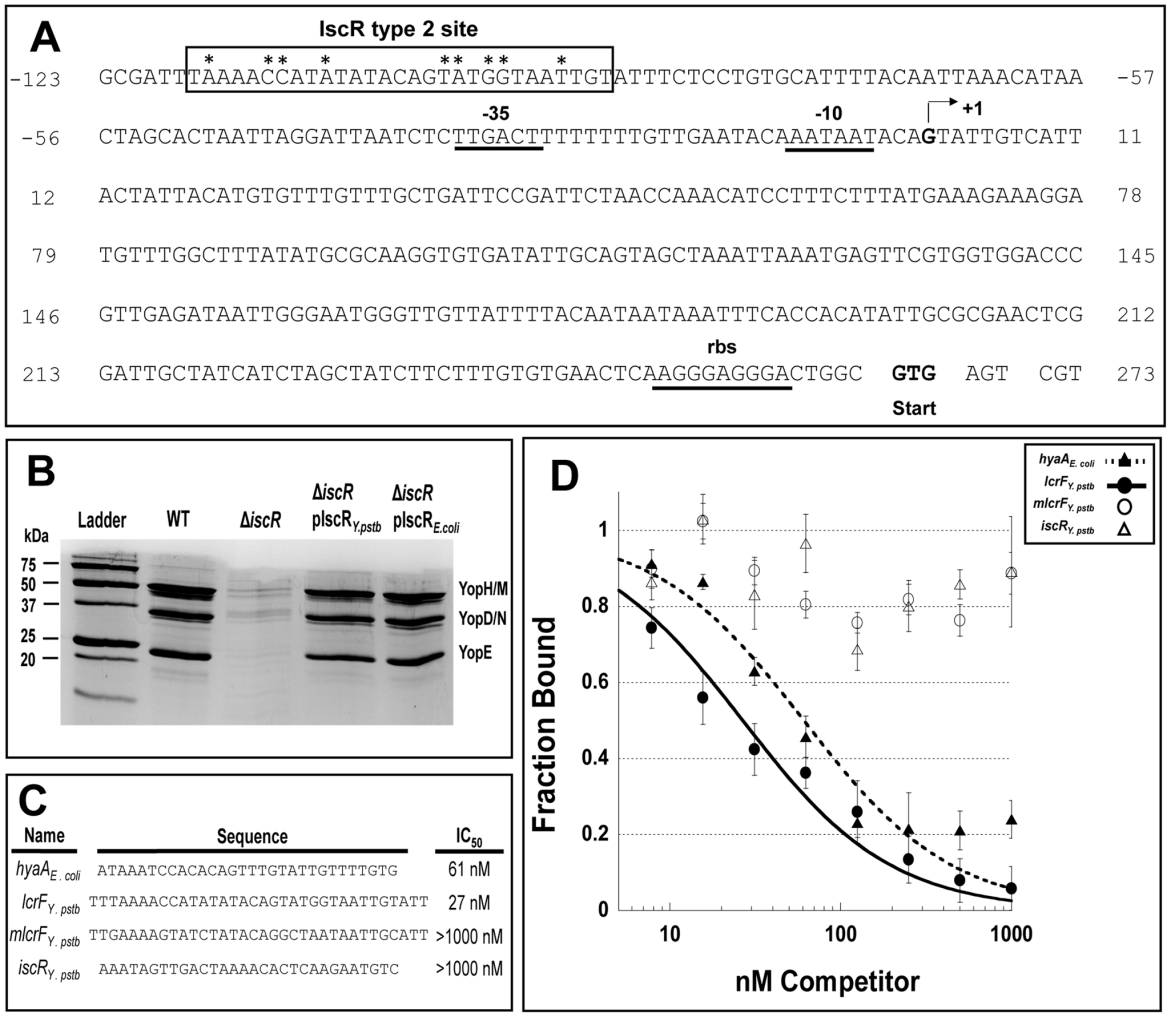
IscR binds a novel motif 2 site within the *lcrF* promoter region. (**A**) Displayed is the promoter region of the *yscW-lcrF* operon including −35 and −10 regions, the transcriptional start site (+1) and the ribosome binding site (RBS) [24]. The IscR type 2 DNA-binding site is indicated by a black box. The nine bases previously found to be important for IscR binding are indicated by asterisks [35]. (**B**) *Y. pseudotuberculosis* IP2666 wild type (WT), *iscR* deletion (Δ*iscR*), Δ*iscR* complemented with *Y. pseudotuberculosis iscR* (Δ*iscR* pIscR_Y.pstb_), and Δ*iscR* complemented with *E. coli iscR* (Δ*iscR* pIscR_E.coli_) strains were grown in 2xYT low calcium media at 37°C to induce type III secretion in the absence of host cells. Proteins in the bacterial culture supernatant were precipitated and visualized alongside a protein molecular weight marker (Ladder) on a polyacrylamide gel using commassie blue. Sample loading was normalized for OD_600_ of each culture. These results are representative of three independent experiments. (**C**) The competitor DNA sequences used for the competition assay and the resulting IC_50_ concentrations are displayed. Nucleotides in bold and underlined correspond to those that were changed in the *mlcrF* sequence and have been found to be important for IscR binding in *E. coli* [33]. (**D**) Competition assay utilizing 59 nM *E. coli* apo-locked IscR (IscR-C92A) and 5 nM TAMRA labeled *hya* DNA [33]. Assay were performed using a range of 8 to 1000 nM unlabeled competitor DNA, including the known *E. coli hya* site competitor (closed triangles), the *in silico* identified *Y. pseudotuberculosis lcrF* site competitor (closed circles), mutated *lcrF* (*mlcrF*) site competitor (open circles), and the negative control *Y. pseudotuberculosis isc in silico* identified motif I site competitor (open triangles). Shown are the averages ± SEM from three independent experiments.

To determine whether IscR might directly regulate T3SS gene expression, we carried out bioinformatic analysis to search pYV for sequences resembling the *E. coli* IscR type 2 motif (xxWWWWCCxYAxxxxxxxTRxGGWWWWxx) [30], [31], [33], as the DNA-binding domain of *Yersinia* IscR is 100% identical to that of *E. coli* IscR (Figure 1A). We searched within the 150 nucleotides upstream of the 99 genes encoded on the pYV plasmid and obtained a ranked list of putative type 2 motifs (data not shown). Among these was a site located within the *yscW-lcrF* promoter region (Figure 8A) [24]. To test whether IscR bound specifically to this site, we performed equilibrium DNA competition assays utilizing purified *E. coli* IscR-C92A (apo-locked IscR) [33], with a fluorescently-labeled *E. coli hya* type 2 site previously identified by Nesbit et al. [33]. Purified *E. coli* IscR was utilized in this assay, as complementation of the *Y. pseudotuberculosis* Δ*iscR* mutant strain with IscR of *E. coli* encoded on a plasmid fully restored secretion of T3SS cargo (Figure 8B). Competitor DNA included unlabeled *E. coli hya* as a positive control, the identified site within the *Yersinia yscW-lcrF* promoter region, a mutated version of this sequence (*mlcrF*), where nucleotides previously demonstrated in *E. coli* to be important for type 2 motif binding were altered [33], as well as one of the *Y. pseudotuberculosis isc* type 1 motif sites we identified as a negative control (Figure S2B & Figure 8C). We found that unlabeled *lcrF* DNA competed as well as unlabeled *hya* DNA (IC_50_ 27 nm and 61 nm, respectively), suggesting that IscR can indeed bind to the identified type 2 motif upstream of *lcrF* (Figure 8D). Furthermore, mutation of key nucleotides in the *lcrF* promoter sequence led to alleviation of competition and increased the IC_50_ to greater than 1000 nM, a level comparable to that of the *isc* negative control type 1 motif site (Figure 8D). These findings suggest that IscR may regulate transcription of the T3SS through a type 2 motif within the *yscW-lcrF* promoter region.

## Discussion

In this study, we present the first characterization of the iron-sulfur cluster regulator, IscR, of *Yersinia*. Initially identified through a genetic screen for modulators of Ysc T3SS function, *iscR*-deficient *Y. pseudotuberculosis* had a dramatic defect in secretion of T3SS effector proteins and in targeting macrophages through their T3SS, yet displayed normal growth in broth culture and wild type flagellar motility. Bioinformatic and DNA binding analysis revealed an IscR binding site upstream of the operon encoding the T3SS master regulator LcrF, indicating that IscR controls expression of the Ysc T3SS. Collectively, these findings indicated that IscR is a central component of the *Y. pseudotuberculosis* T3SS regulatory cascade.

**Figure 9:**
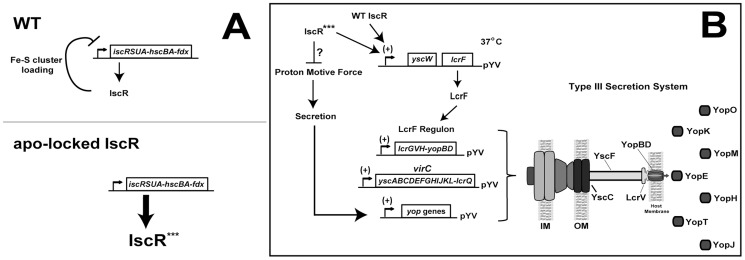
Regulation of the *isc* and *lcrF* operons by IscR. (**A**) Model of *isc* operon transcriptional control in the *Y. pseudotuberculosis* wild type and apo-locked IscR strains based on previous work on *E. coli* IscR [32], [34] and on data shown here. In wild type bacteria, the Isc Fe-S cluster biogenesis pathway loads a [2Fe-2S] cluster onto IscR (holo-IscR) [32], which recognizes a type 1 DNA-binding motif in the *isc* promoter to repress transcription in a negative feedback loop. Expression of the apo-locked IscR allele (***, IscR-C92A/C98A/C104A) results in loss of holo-IscR-mediated repression, thereby increasing transcription of the *isc* operon relative to wild type, resulting in a 30-fold increase in *iscR*. (**B**) Model depicting the mechanism by which IscR controls the *Y. pseudotuberculosis* Ysc T3SS. Holo- and apo-IscR are predicted to bind a newly identified type 2 DNA-binding site within the *yscW-lcrF* operon encoding the LcrF T3SS master regulator. Subsequently, LcrF expression leads to transcription of the LcrF regulon, which includes the *lcrGVH-yopBD* and *virC* operons and *yop* genes [17], [20], [22], [53], [54]. These genes encode the majority of T3SS structural, regulatory, and effector proteins. However, through an as yet undefined mechanism, overexpression of apo-locked IscR leads to a decrease in the proton motive force, which is required for type III secretion [50]. As Yop secretion positively regulates *yop* gene transcription [51], [52], the secretion defect of the apo-locked IscR mutant is predicted to lead to a decrease in effector *yop* transcription.

Both *E. coli* holo- and apo-IscR are active transcription factors with distinct DNA binding targets. Holo-IscR can bind both type 1 and 2 motifs whereas apo-IscR can only bind type 2 motifs. IscR of *E. coli* autoregulates the *isc* operon, *iscRSUA-hscBA-fdx*, through binding to type 1 motifs within the *isc* promoter region [34]. In addition, Giel et al. described increased transcription of the genes located immediately downstream of the *isc* operon, *yfhJ*-*pepB*-*sseB,* in an *iscR* mutant, suggesting a negative regulatory effect on these genes as well [30]. We observed derepression of the *iscRSUA-hscBA-fdx* operon and the *yfhJ*-*pepB*-*sseB* locus in the *Y. pseudotuberculosis* Δ*iscR* mutant as well as the mutant expressing apo-locked IscR. Furthermore, we identified two sites within the *Y. pseudotuberculosis isc* promoter that closely match the *E. coli* IscR motif I consensus sequence. These data indicate that the *iscRSUA-hscBA-*fdx operon, and possibly the *yfhJ*-*pepB*-*sseB* locus, are negatively regulated by holo-IscR in *Yersinia* as they are in *E. coli* (Figure 9A). IscR in *E. coli* is known to activate transcription of the *sufABCDSE* operon through binding to a type 2 motif [29]. Our analysis revealed that the *Y. pseudotuberculosis* apo-locked IscR mutant overexpresses the *sufABCDS* operon compared to the wild type and Δ*iscR* strains, which we predict results from the overexpression of IscR observed in the apo-locked mutant as found in *E. coli* [32], [33]. We identified a site within the *Y. pseudotuberculosis suf* promoter region that closely resembles an *E. coli* IscR type 2 motif (data not shown). Together, these data indicate that the *suf* operon is positively regulated by IscR in *Yersinia* as in *E. coli*. Thus, we propose that IscR of *Y. pseudotuberculosis* modulates transcription of both the *isc* and *suf* Fe-S cluster biosynthesis pathways via mechanisms established for its *E. coli* ortholog.

In addition to control of Fe-S cluster biogenesis pathway expression, we present evidence that IscR controls expression and function of the *Y. pseudotuberculosis* T3SS. Bioinformatic analysis revealed a type 2 motif within the promoter of the T3SS master regulator LcrF that contained all nine bases previously found to be important for IscR binding (Figure 8A) [33]. Indeed, DNA binding assays demonstrated that IscR is able to specifically recognize this type 2 motif, suggesting that IscR may be acting directly to promote transcription of *lcrF* (Figure 9B). In support of this, we observed a marked decrease in transcription of numerous T3SS genes in the Δ*iscR* mutant strain. These include the gene that encodes LcrF, as well as a number of LcrF-regulated genes including the *virC* operon, *yopK*, *yopT*, *yopM*, *yopH*, *yopJ*, and *lcrGVH-yopBD* [17], [20], [22], [53], [54]. The *lcrF* type 2 motif is further upstream of the -10/-35 region previously identified by Böhme et al. [24] than other IscR binding sites that promote transcription [33], as we propose this site does. However, there may be an alternative −10/−35 region closer to the identified motif 2 site that might be used under specific growth conditions. Together, these data suggest that IscR is required for full expression of *lcrF* and LcrF-regulated genes through binding to a type 2 motif in the *yscW-lcrF* promoter (Figure 9B).

Based on these findings, an IscR mutant unable to coordinate an Fe-S cluster (apo-locked IscR) should lead to restoration of T3SS expression. Indeed, transcription of the *yscW-lcrF* and *virC* operons, as well as the majority of genes in the *lcrGVH-yopBD* operon, were no longer significantly decreased in the apo-locked IscR mutant compared to the Δ*iscR* strain. However, decreased transcription of *yopE*, *yopK*, *yopM*, and *yopH* as well as a severe defect in secretion of Yops was still observed. This could be explained by a deficiency in the apo-locked mutant's membrane potential, but not in the Δ*iscR* strain (Figure 9B). Wilharm et al., demonstrated that *Y. enterocolitica* motility and type III secretion requires the proton motive force [50]. Indeed, the apo-locked *Y. pseudotuberculosis* strain displayed a significant motility defect while the Δ*iscR* mutant was fully motile. Therefore, the type III secretion defect of the *Y. pseudotuberculosis* apo-locked IscR mutant can be explained by a deficiency in the proton motive force. Furthermore, the defect in YopHEMK transcription in the apo-locked IscR mutant may be explained by the fact that Yop secretion has a positive regulatory effect on Yop transcription [51], [52]. Together, these data suggest that apo-IscR can promote LcrF transcription, but that locking iscR is the apo form causes a proton motive force defect that prevents effector Yop transcription and secretion (Figure 9B).

It is unclear why locking IscR in the apo-locked form leads to a proton motive force defect. We observed ∼9-fold more *suf* transcript in the apo-locked IscR mutant compared to the Δ*iscR* strain that does not have a proton motive force defect, whereas the *isc* operon was expressed to the same degree in both mutants. Ezraty et al. recently showed that expression of the *suf*, but not the *isc*, operon in *E. coli* leads to a proton motive force defect, possibly as a result of impaired loading of Fe-S clusters into aerobic respiratory complexes [55]. Although the *isc* operon is expressed in the apo-locked *Y. pseudotuberculosis* mutant, perhaps overexpression of the *suf* pathway leads to misassembly of the Fe-S complexes of the electron transport chain that drive the proton motive force.

Both holo- and apo-IscR are predicted to bind to the type 2 motif within the *yscW-lcrF* promoter [33]. Based on previous data on *E. coli* IscR [28]–[30], [34], [56], low iron, aerobic growth, or high oxidative stress conditions are predicted to result in high expression of IscR through derepression of the *isc* operon, which in turn should increase T3SS gene expression. Likewise, high iron, anaerobic, or low oxidative stress conditions should lead to decreased IscR levels and therefore lower T3SS expression. Under normal aerobic culture conditions, we do not observe a change in wild type *Y. pseudotuberculosis* type III secretion when iron levels are altered (data not shown). However, *in vivo*, bacteria may be present in microaerophilic or anaerobic niches, where changes in iron bioavailability and reactive oxygen species production may impact *iscR* and T3SS gene expression. Upon ingestion by a host animal, *Y. pseudotuberculosis* enters the lumen of the intestine, which receives approximately 15 mg of iron per day [57], [58]. In the small intestine, *Y. pseudotuberculosis* can cross the gut barrier and enter the bloodstream and deeper tissues, which have very low iron bioavailability (∼10^−24^ M free serum iron) [59]–[61]. Sequestration of iron by iron carriers in mammalian tissues is an important host defense mechanism to prevent growth of bacterial pathogens, the majority of which require iron for growth [62]. The Ysc T3SS has been shown to be required for *Y. pseudotuberculosis* pathogenesis in these deep tissue sites that are low in iron bioavailability [44]. Perhaps *Y. pseudotuberculosis* uses IscR to sense iron, O_2_, and/or ROS concentration in order to optimally control T3SS expression *in vivo*.

Consistent with the severe T3SS expression defect displayed by the *Y. pseudotuberculosis* Δ*iscR* strain, this mutant was deficient in colonization of the Peyer's patches, spleen, and liver. Interestingly, the Δ*iscR* mutant was also defective in colonization of the mesenteric lymph nodes (MLN), yet T3SS mutants were previously shown to persist in the MLN and chromosomally-encoded factors were found to be important for *Y. pseudotuberculosis* survival in this tissue [24], [63], [64]. These results indicate that the virulence defect of the *Y. pseudotuberculosis* Δ*iscR* strain may not be due solely to misregulation of the T3SS, suggesting the existence of other IscR gene targets important for virulence. IscR of *Pseudomonas aeruginosa* has been shown to be important for full virulence through its ability to upregulate KatA, encoding a catalase that protects against oxidative stress [38], [65]–[67]. In *Vibrio vulnificus,* IscR upregulates two genes encoding the antioxidants peroxiredoxin and glutaredoxin 2, and is essential for survival during exposure to reactive oxygen species [40]. Interestingly, our analysis suggests that *Y. pseudotuberculosis* IscR plays an opposite regulatory role, as IscR negatively affects expression of the genes encoding cellular detoxification proteins KatY, Tpx, SodC and SodB. Furthermore, hydrogen peroxide sensitivity assays showed comparable levels of survival between the *Y. pseudotuberculosis* wild type and Δ*iscR* strains (Figure S5). This suggests that the virulence defect observed for the Δ*iscR Y. pseudotuberculosis* mutant is not due to increased susceptibility to oxidative stresses encountered during infection. Pathways other than the T3SS, such as the *hmu* hemin uptake system, were found to be misregulated in the *Y. pseudotuberculosis* Δ*iscR* strain (Table 2 & Figure 4B). While the *hmu* operon was shown to not affect *Y. pestis* virulence, it is possible that IscR control of the *Y. pseudotuberculosis hmu* pathway is important for virulence.

In summary, we present the first characterization for the iron-sulfur cluster regulator, IscR, of *Yersinia*. We reveal that IscR regulates genes involved in Fe-S cluster assembly in a manner akin to that of *E. coli*. Most notably, we demonstrate that mutation of IscR leads to decreased function of the *Y. pseudotuberculosis* T3SS and that this is due to a decrease in transcription of genes encoding structural, regulatory, and effector proteins. Furthermore, we present evidence showing that IscR is essential for the virulence of *Y. pseudotuberculosis* and that this attenuation is likely due, in part, to direct regulation of the T3SS by IscR. Collectively, this study argues for the important and novel role of IscR in the virulence of *Y. pseudotuberculosis* as well as regulation of the Ysc T3SS, and identifies IscR as a potential target for novel antimicrobial agents.

## Materials and Methods

All animal use procedures were in strict accordance with the NIH Guide for the Care and Use of Laboratory Animals and were approved by the UCSC Institutional Animal Care and Use Committee.

### Bacterial strains, plasmids and growth conditions

All strains used in this study are listed in Table 3. *Y. pseudotuberculosis* strains were grown in either 2xYT or M9 minimal media supplemented with casamino acids [68], referred to here as M9, at 26°C with shaking at 250 rpm, unless otherwise indicated. Where stated, Yop synthesis was induced via back-dilution of cultures into either M9 or low calcium media (2xYT plus 20 mM sodium oxalate and 20 mM MgCl_2_) to an OD_600_ of 0.2 and grown for 1.5 h at 26°C/shaking followed by 2 h at 37°C/shaking as previously described [69].

**Table 3:** *Y. pseudotuberculosis* strains used in this study.

Strain	Background	Mutation(s)	Reference
WT	IP2666	Naturally lacks full-length YopT	[47]
Δyop6	IP2666	Δ*yopHEMOJ*	[43]
Δ*yscNU*	IP2666	Δ*yscNU*	[63]
pYV^−^	IP2666	Δ*yscBL* pYV cured	[43]
Δyop6/Δ*yopB*	IP2666	Δ*yopHEMOJ* Δ*yopB*	[43]
Δyop6/*flhD_Y.pestis_*	IP2666	Δ*yopHEMOJ* inactive, *Y. pestis* allele of *flhD*	[43]
Δyop6/Tn1	IP2666	Δ*yopHEMOJ iscR*_89bp_::Tn*Himar1*	This work
Δyop6/Tn2	IP2666	Δ*yopHEMOJ iscR*_281bp_::Tn *Himar1*	This work
Δyop6/Δ*iscR*	IP2666	Δ*yopHEMOJ* Δ*iscR*	This work
Δ*iscR*	IP2666	Δ*iscR*	This work
Δ*iscR* pIscR	IP2666	Δ*iscR* pACYC184::*iscR* ^+^	This work
apo-IscR	IP2666	IscR-C92A/C98A/C104A	This work
apo-IscR pIscR	IP2666	IscR-C92A/C98A/C104A pACYC184::*iscR* ^+^	This work

### Construction of *Y. pseudotuberculosis* mutant strains

The *iscR* deletion mutant (Δ*iscR*) was generated via splicing by overlap extension PCR [70]. Primer pairs F5'iscR/R5'iscR and F3'iscR/R3'iscR (Table S3), designed using MacVector and Primer 3 software (http://fokker.wi.mit.edu/primer3/input.htm), were used to amplify ∼500 bp 5′ and 3′ of the *iscR* coding region, respectively. Amplified PCR fragments served as templates in an overlap extension PCR using the outside primers F5'iscR and R3'iscR.

The IscR-C92A/C98A/C104A mutant (apo-IscR) was generated via splicing by overlap extension PCR [70]. Primer pairs F5'apo-IscR/R5'apo-IscR and F3'apo-IscR/R3'apo-IscR (Table S3), designed using MacVector and Primer 3 software (http://fokker.wi.mit.edu/primer3/input.htm), were used to amplify ∼500 bp 5′ and 3′ within the *iscR* coding region, respectively. Amplified PCR fragments served as templates in an overlap extension PCR using the outside primers F5'apo-IscR and R3'apo-IscR. Nucleotide changes within the internal primers R5'apo-IscR and F3'apo-IscR allowed for amplification of *iscR* target containing sequences coding for alanine substitutions of the three conserved cysteines that coordinate an Fe-S cluster.

The resulting ∼1 kb fragments were cloned into the TOPO TA cloning vector (Invitrogen) and further subcloned into a BamHI- and NotI-digested pSR47s suicide plasmid (λpir-dependent replicon, kanamycin^R^ (Kan^R^), *sacB* gene conferring sucrose sensitivity) [71], [72]. Recombinant plasmids were transformed into *E. coli* S17-1 λpir competent cells and later introduced into *Y. pseudotuberculosis* IP2666 via conjugation. The resulting Kan^R^, irgansan^R^ (*Yersinia* selective antibiotic) integrants were grown in the absence of antibiotics and plated on sucrose-containing media to select for clones that had lost *sacB* (and by inference, the linked plasmid DNA). Kan^S^, sucrose^R^, congo red-positive colonies were screened by PCR and subsequently sequenced to verify loss of the intended *iscR* coding region.

The *iscR* complement construct was generated by insertion of a fragment containing the *iscR* coding region as well as 530 bp of 5′ upstream sequence. This was PCR amplified using primer pair FiscRC and RiscRC, and cloned into the vector pACYC184 via BamHI/SalI restriction sites [73], [74]. Recombinant plasmids were transformed into *E. coli* S17-1 λpir competent cells and later introduced into *Y. pseudotuberculosis* IP2666 Δ*iscR* via a modified transformation method [75]. Briefly, recipient *Yersinia* strains were grown overnight in LB containing 2% glucose at 26°C. Cultures were centrifuged at 3,500 rpm for 3 min then washed with 750 µl of ice-cold sterile diH_2_O and repeated for a total of three washes. Washed pellets were resuspended in 100 µl of sterile diH_2_O, combined with 3 µl of plasmid and electroporated at EC2. Cells were allowed to recover in 1 mL SOC media for 1 h at 26°C followed by plating on LB containing carbenicillin to select for *Yersinia* bearing the plasmid of interest. Clones were confirmed by PCR analysis, using a combination of gene- and vector-specific primers, to construct both the Δ*iscR* complemented strain (Δ*iscR* pIscR) and the apo-IscR complemented strain (apo-IscR pIscR).

The nonmotile Δyop6/*flhDC^Y.pestis^* mutant was generated by crossing in the *Y. pestis flhDC* gene into *Y. pseudotuberculosis*. *Y. pestis flhD* has a frameshift mutation, resulting in suppression of flagellin production [76]. The suicide plasmid pSB890 encoding a partial *flhC* gene and the full *flhD* gene from the *Y. pestis* KIM strain, generously provided by Dr. Brad Cookson, was conjugated into *Y. pseudotuberculosis* Δyop6 and nonmotile, recombinant mutants isolated as previously described [46].

### Transposon screen generation and insertion site identification

Transposon mutagenesis was preformed similarly to Crimmins et al. [64]. Briefly, *E. coli* SM10λpir harboring pSC189, which encodes Himar1 [77], was mated with *Y. pseudotuberculosis* Δyop6. Mating culture was then pelleted, resuspended, spread out evenly among six 150 mm×15 mm petri plates containing LB supplemented with 2 µg mL^−1^ irgasan and 30 µg mL^−1^ Kan, and incubated for 3 days at room temperature. Colonies were patched onto LB supplemented with 100 µg mL^−1^ carbenicillin to ensure insertion of the transposon. Colony patches were used to grow 2xYT overnight cultures in 96-well plates, which were then frozen down to preserve the library. HEK293T cells were plated in 96-well white clear bottom plates (Corning) and transfected with a plasmid encoding a luciferase reporter gene fused to an NFκB-dependent promoter (Stratagene). Mutants from the transposon library were grown overnight in 96 well plates in M9 at 26°C and used to infect the transfected HEK293T cell monolayers. After 4 h incubation at 37°C, 100 µl of 1∶1 NeoLite:PBS solution was added to each well of the 96-well clear-bottom white plate (Corning), and luminescence was measured using a Victor^3^ plate reader (PerkinElmer). Each transposon mutant was assayed in duplicate. The positions of the transposons in the *iscR*::Tn1 and *iscR*::Tn2 mutants were determined by plasmid rescue, as previously described [78], except BamHI was used for digestion of genomic DNA.

### NFκB activity assay

Validation of the transposon screen was performed through the use of an NF_Κ_B activity assay, which is based on our previous work showing that *Y. pseudotuberculosis* induces NFκB activation in HEK293T cells dependent on expression of a functional T3SS [43]. Briefly, HEK293T cells were transfected with a plasmid encoding a luciferase reporter gene fused to an NFκB-dependent promoter (Stratagene). Bacterial strains were grown overnight in 2xYT and subcultured to an OD_600_ of 0.2 into low calcium media and grown at 26°C for 1.5 h followed by a shift to 37°C for an additional 1.5 h to induce the T3SS. Bacterial cultures were resuspended in prewarmed (37°C) DMEM and 200 µl aliquots were then used to infect the HEK293T cells containing the luciferase reporter plasmid at an MOI of 10. After 4 h incubation at 37°C, 100 µl of 1∶1 NeoLite:PBS solution was added to each well of the 96-well clear-bottom white plate (Corning), and luminescence was measured using a Victor^3^ plate reader (PerkinElmer). Data from three separate wells were averaged for each independent experiment.

### Type III secretion assay

Visualization of T3SS cargo secreted in broth culture was performed as previously described [46]. Briefly, *Y. pseudotuberculosis* in M9 low calcium media (M9 plus 20 mM sodium oxalate and 20 mM MgCl_2_) was grown for 1.5 h at 26°C followed by growth at 37°C for 2 h. Cultures were normalized by OD_600_ and pelleted at 13,200 rpm for 10 min at room temperature. Supernatants were removed and proteins precipitated by addition of trichloroacetic acid (TCA) at a final concentration of 10%. Samples were incubated on ice for 20 min and pelleted at 13,200 rpm for 15 min at 4°C. Resulting pellets were washed twice with ice-cold 100% EtOH and subsequently resuspended in final sample buffer (FSB) containing 20% dithiothreitol (DTT). Samples were boiled for 5 min prior to running on a 12.5% SDS PAGE gel.

### Ethidium bromide entry assay

Evaluation of pore formation was performed via the ethidium bromide (EtBr) entry assay as previously described [46]. Briefly, 2×10^4^ immortalized C57Bl/6 BMDMs were plated in a 96 well clear bottom black plate (Corning) in 100 uL DMEM +10% FBS. Infection was performed in triplicate at an MOI of 25. Plates were centrifuged at 750×g at 4°C for 5 min to facilitate contact. Infections were carried out at 37°C with 5% CO_2_ for 2 h, at which point media was aspirated and replaced with 30 µL of PBS containing 25 µgmL^−1^ ethidium bromide (EtBr) and 12.3 µg mL^−1^ Hoechst dye. The cell monolayer was visualized using an ImageXpressMICRO automated microscope and MetaXpress analysis software (Molecular Devices). The percent of EtBr-positive cells was calculated by dividing the number of EtBr-stained cells by the number of Hoechst-stained cells. Data from three separate wells was averaged for each independent experiment.

### Growth curves

*Y. pseudotuberculosis* strains were cultured overnight in 2xYT or M9 at 26°C and sub-cultured to an OD_600_ of 0.2 in 25 mL of either 2xYT or M9. Cultures were incubated at either 26°C or 37°C with shaking at 250 rpm and optical density measured at 600 nm every hour for 9 h.

### Mouse infections

All animal use procedures were in strict accordance with the NIH *Guide for the Care and Use of Laboratory Animals* and were approved by the UC Santa Cruz Institutional Animal Care and Use Committee. Eleven to twelve-week-old 129S6/SvEvTac mice from our breeding facilities were used for oral infections as previously described [79]. Briefly, mice were orogastrically inoculated with 2×10^8^ CFU in a 200 µl volume using a feeding needle. Mice were given food and water *ad libitum* and were euthanized at 5 days post-inoculation. Peyer's patches, mesenteric lymph nodes, spleens, and livers were isolated and homogenized for 30 s in PBS followed by serial dilution and plating on LB supplemented with 1 µg mL^−1^ irgasan for CFU determination.

### RNAseq analysis

RNA was isolated from the IP2666 wild type and isogenic Δ*iscR* and apo-IscR strains grown for 3 h at 37°C in M9, using the RNeasy Mini Kit (Qiagen) as per the manufacturer's protocol. We chose M9 media for our RNASeq analysis because this condition enables expression of T3SS genes and secretion of T3SS cargo at 37°C [68]. Contaminating DNA was removed from the RNA samples using a DNA-free kit (Life Sciences). Samples were subjected to removal of contaminating rRNA via the Ribo-Zero Magnetic Kit for Gram-negative bacteria (Epicentre). The cDNA library was created using the NEBNext Ultra Directional RNA Library Prep Kit for Illumina (NEB). These studies were performed with three biological replicates per condition. Six indexed samples were sequenced per single lane using the HiSeq2500 Illumina sequencing platform for 50 bp single reads (UC Davis Genome Center) and subsequently analyzed and visualized via the CLC Genomics Workbench version 5.5.1 (CLC bio). Samples were normalized for both sequence depth and gene size by determining RPKM (Reads Per Kilo base per Million reads) and mapped to the *Y. pseudotuberculosis* genome (IP32953). Differentially regulated genes were identified as those displaying a fold change with an absolute value of 2 or greater. Statistical significance was determined by baySeq test with a corrected FDR post hoc test where p<0.05 was deemed significant [80].

### Real-time PCR

Total RNA generated from our RNAseq analysis at a concentration of 2 µg was used to make cDNA as previously described [81]. SYBR Green PCR master mix (Applied Biosystems) was used for qPCR reactions according to the manufacturer's instructions and a 60°C annealing temperature. Primers used are listed in Table S4. Control primers were for the 16S rRNA as described previously [82]. Results were analyzed using the Bio-Rad CFX software.

### Virulence plasmid map generation

Average RPKM values generated from RNAseq analysis for the wild type, Δ*iscR* and apo-IscR mutants were converted to log_2_-ratios (log_2_(RPKM_mutant_/RPKM_wt_) for each gene encoded on the virulence plasmid, pYV. These values were converted to a Circos heatmap [83] and plotted against the respective pYV base coordinate positions from *Y. pseudotuberculosis* IP32953.

### Motif identification and search

Position specific scoring matrix (PSSM) was generated by the alignment of the known *E. coli* IscR type 2 motifs (Table S4) (Maverix Biomics, Inc) [31]. PSSM of type 2 was used to scan against the 150-nt upstream of 99 genes encoded on the *Y. pseudotuberculosis* pYV plasmid and obtained a ranked list of putative type 2 motifs.

### DNA binding fluorescence anisotropy assays

Fluorescence anisotropy was measured similar to Nesbit et al., [33]. *E. coli* apo-IscR lacking the [2Fe-2S] cluster (IscR-C92A) was isolated anaerobically following the protocol described previously for wild type IscR [30]. Competition assays were performed using 5nM of 30-mer dsDNA of the known *E. coli hyaA* type 2 motif containing a 5′ TAMRA fluorophore (IDT) on the top strand and unlabeled competitor dsDNA concentrations ranged from 8 to 1000 nM (IDT, Table S4). DNA was annealed by heating equimolar concentrations of complementary DNA strands in annealing buffer (40 mM Tris (pH 7.9), 30 mM KCl) to 95°C for 5 min followed by slow cooling to room temperature over 2 hours. Annealed DNA was incubated with 90 nM apo-IscR in anisotropy buffer (40 mM Tris pH 7.9, 150 mM KCl, 100 ng ul^−1^ Salmon Sperm DNA) for 12 min at room temperature and anisotropy was measured using an EnVision 2103 Multilabel Reader (Perkin Elmer) with Wallac EnVision Manager software. Data is representative of experiments performed on three separate days.

### Motility assay

Motility was analyzed by spotting 1 µl aliquots of either a nonmotile strain bearing an inactive, *Y. pestis* allele of *flhD* (Δyop6/*flhD^Y.pestis^*), WT, Δ*iscR*, or apo-IscR strains onto motility agar plates (1% tryptone, 0.25% agar) from overnight cultures standardized to an OD_600_ of 2.5. Plates were incubated at room temperature for 1 day, at which point the diameters of the colonies were determined and used to calculate percent motility relative to WT, which was set at 100%.

### Measurement of the membrane potential

The electrical potential was measured similar to the JC-1 red/green dye assay previously described for *E. coli* [84]. JC-1 is a membrane-permeable dye that emits green fluorescence (∼530 nm) upon excitation when the dye is in the monomeric form. Due to the membrane potential of the bacterial cell, JC-1 dye will form J aggregates which emit red fluorescence (∼590 nm). If the membrane potential decreases, there will be a decrease in J aggregate formation and subsequently a decrease in red fluorescence. As such, membrane potential can be displayed as a ratio of red/green fluorescence. Briefly, *Y. pseudotuberculosis* wild type and isogenic Δ*iscR*, Δ*iscR* complemented (Δ*iscR* pIscR), apo-IscR and apo-IscR complemented (apo-IscR pIscR) strains were grown overnight in M9 at 26°C. Strains were subcultured to an OD_600_ of 0.2 in M9 and grown at 37°C for 3 hours. A negative control containing a sample of wild type *Y. pseudotuberculosis* treated with 40 µM of the protonophore, CCCP (carbonyl cyanide m-chlorophenylhydrazone), during the last 30 min of growth was included in each experiment. After incubation at 37°C, 1 mL aliquots were harvested for each strain and pelleted at 4,500×g for 3 min followed by resuspension in 1 mL of permeabilisation buffer (10 mM Tris-HCl, pH 7.6, 1 mM EDTA and 10 mM glucose). Post resuspension, 2 µl of the membrane-permeable JC-1 dye (5 mg/mL) was added and the samples were incubated at room temperature for 30 min. Samples were then pelleted at 4,500×g for 3 min and resuspended in 500 µl of permeabilisation buffer. Slides were prepared by first coating with Poly-L-Lysine solution through addition of 100 µl aliquots of a 0.01% solution followed by a 5 min incubation at room temperature. Slides were washed a total of 3 times with sterile diH_2_O. Once dry, 10 µl of prepared sample was added to the slide and allowed to adhere for 5 min. Unattached bacteria were removed by washing with PBS and excess liquid removed via aspiration. A coverslip was applied and the cells were imaged using a LSM 5 PASCAL laser scanning microscope (Zeiss) fitted with a Plan-Apochromat 63x/1.4 Oil DIC objective and analyzed using the LSM 510 software (Zeiss). Quantification of image intensities was performed using ImageJ [85].

## Supporting Information

Figure S1 **IscR does not affect** ***Y. pseudotuberculosis*** **growth under non-T3SS-inducing conditions, but partially alleviates T3SS-associated growth restriction.** The *Y. pseudotuberculosis* WT, Δ*iscR*, apo-IscR and, where applicable, Δ*iscR* and apo-IscR complemented strains (Δ*iscR* pIscR and apo-IscR pIscR, respectively) and *Y. pseudotuberculosis* lacking the virulence plasmid pYV (pYV^−^), were grown (**A**) in M9 at 37°C, (**B**) in 2xYT at 37°C, (**C**) in M9 at 26°C or (**D**) in 2xYT at 37°C. Optical density of the cultures were monitored at 600 nm every hour for 9 h. The averages ± SEM from three independent experiments are shown. * p<0.05, **p<0.01, ***p<0.001 as determined by a Student *t* test relative to the wild type.
(TIF)

Figure S2 **Deletion of IscR leads to increased transcription of Fe-S cluster biogenesis genes.** (**A**) RPKM expression levels generated from RNAseq analysis of *Y. pseudotuberculosis* Δ*iscR* and apo-IscR mutants relative to WT for 12 genes involved in Fe-S cluster biogenesis are displayed. *p<0.001 as determined by Bayseq test with a corrected FDR post hoc test from three independent experiments. (**B**) Displayed is the nucleotide sequence of a region 130 bp upstream of the putative IscR start codon in *Y. pseudotuberculosis* IP 32953 including the putative transcriptional start site (arrow; UCSC Microbial Genome Browser) and putative sigma70 promoter elements (−10) and (−35), as well as the two putative IscR type I binding sites (brackets).
(TIF)

Figure S3 **Mutation of** ***iscR*** **does not affect pYV virulence plasmid yield.** Relative amounts of the virulence plasmid, pYV, were analyzed from standardized cultures of the wild type (WT), *iscR* mutant (Δ*iscR*) and pYV^−^ strains grown in M9 at 37°C for 3 hours through midiprep analysis (Promega) according to the manufacturer's protocol. Plasmid yield was quantified via spectrophotometric analysis (Nanodrop). The data is displayed as µg of plasmid isolated per mL of culture ± SEM and is an average of 3 independent experiments. *p≤0.05, as determined by Student *t* test.
(TIF)

Figure S4 **Expression of the** ***suf*** **operon is increased in the apo-locked IscR mutant strain.** RNAseq analysis was performed on WT, Δ*iscR* and apo-IscR *Y. pseudotuberculosis* strains after growth in M9 at 37°C for 3 h (T3SS-inducing conditions). The data is presented as mean RPKM ± SEM and is an average of 3 independent experiments. ***p≤0.0001, as determined by Bayseq followed by FDR post hoc test.
(TIF)

Figure S5 **IscR is not required for survival post-exposure to hydrogen peroxide stress.** Hydrogen peroxide assays were performed similar to Schiano et al. [87]. *Y. pseudotuberculosis* wild type (WT), Δ*iscR*, and *iscR* complemented (Δ*iscR* pIscR) strains were grown overnight in 2xYT at 26°C. Cultures were standardized to an OD_600_ of 0.1 and grown at 26°C with shaking to mid-log phase, at which point they were diluted 1∶10 into fresh 2xYT. Samples were supplemented with 50 µl of either sterile water (negative control) or hydrogen peroxide to a final concentration of 50 mM. Samples were incubated with shaking at 26°C and CFU determined via serial dilution and plating 10 min after the start of treatment. The data is displayed as percent survival (CFU H_2_O_2_/CFU H_2_O)*100) ± SEM and is an average of 3 independent experiments.
(TIF)

Table S1 **RNAseq RPKM values for wild type** ***Y. pseudotuberculosis*** **and the Δ** ***iscR*** **mutant.**
(XLSX)

Table S2 **Total pYV-encoded genes differentially regulated by IscR, identified by RNAseq analysis.**
(DOCX)

Table S3 ***Y. pseudotuberculosis*** **primers used in this study.**
(DOCX)

Table S4 **Known type 2 DNA-binding sequences used for** ***in silico*** **search.**
(DOCX)
