# Supplementary figures and images for: IscR Is Essential for Yersinia pseudotuberculosis Type III Secretion and Virulence
Source: PLoS Pathog. 2014 Jun 12;10(6):e1004194. doi: 10.1371/journal.ppat.1004194 (PMC4055776; doi:10.1371/journal.ppat.1004194)

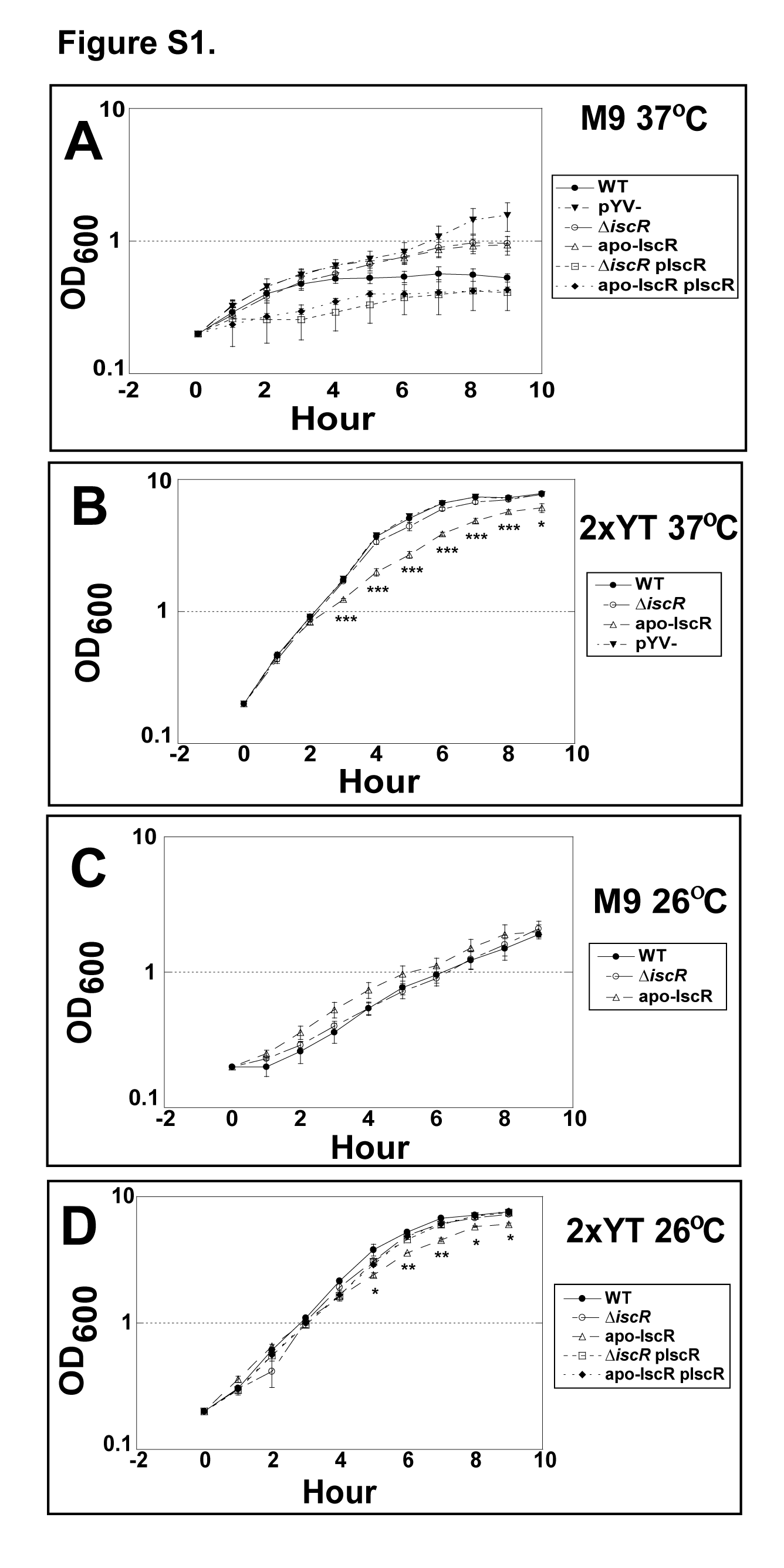

Supplement: Figure S1 — IscR does not affect Y. pseudotuberculosis growth under non-T3SS-inducing conditions, but partially alleviates T3SS-associated growth restriction. The Y. pseudotuberculosis WT, ΔiscR, apo-IscR and, where applicable, ΔiscR and apo-IscR complemented strains (ΔiscR pIscR and apo-IscR pIscR, respectively) and Y. pseudotuberculosis lacking the virulence plasmid pYV (pYV−), were grown (A) in M9 at 37°C, (B) in 2xYT at 37°C, (C) in M9 at 26°C or (D) in 2xYT at 37°C. Optical density of the cultures were monitored at 600 nm every hour for 9 h. The averages ± SEM from three independent experiments are shown. * p<0.05, **p<0.01, ***p<0.001 as determined by a Student t test relative to the wild type. (TIF) [file ppat.1004194.s001.tif]

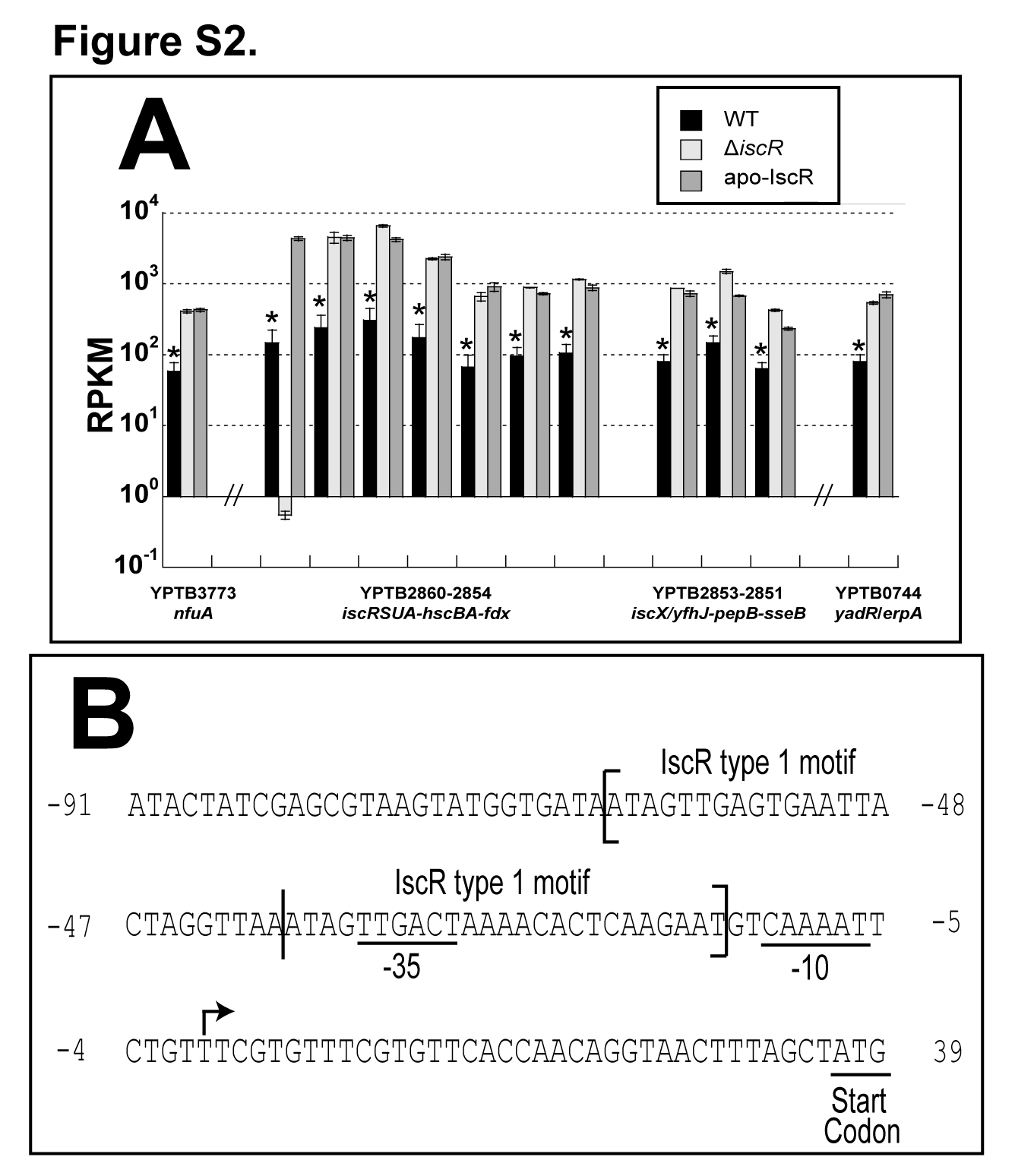

Supplement: Figure S2 — Deletion of IscR leads to increased transcription of Fe-S cluster biogenesis genes. (A) RPKM expression levels generated from RNAseq analysis of Y. pseudotuberculosis ΔiscR and apo-IscR mutants relative to WT for 12 genes involved in Fe-S cluster biogenesis are displayed. *p<0.001 as determined by Bayseq test with a corrected FDR post hoc test from three independent experiments. (B) Displayed is the nucleotide sequence of a region 130 bp upstream of the putative IscR start codon in Y. pseudotuberculosis IP 32953 including the putative transcriptional start site (arrow; UCSC Microbial Genome Browser) and putative sigma70 promoter elements (−10) and (−35), as well as the two putative IscR type I binding sites (brackets). (TIF) [file ppat.1004194.s002.tif]

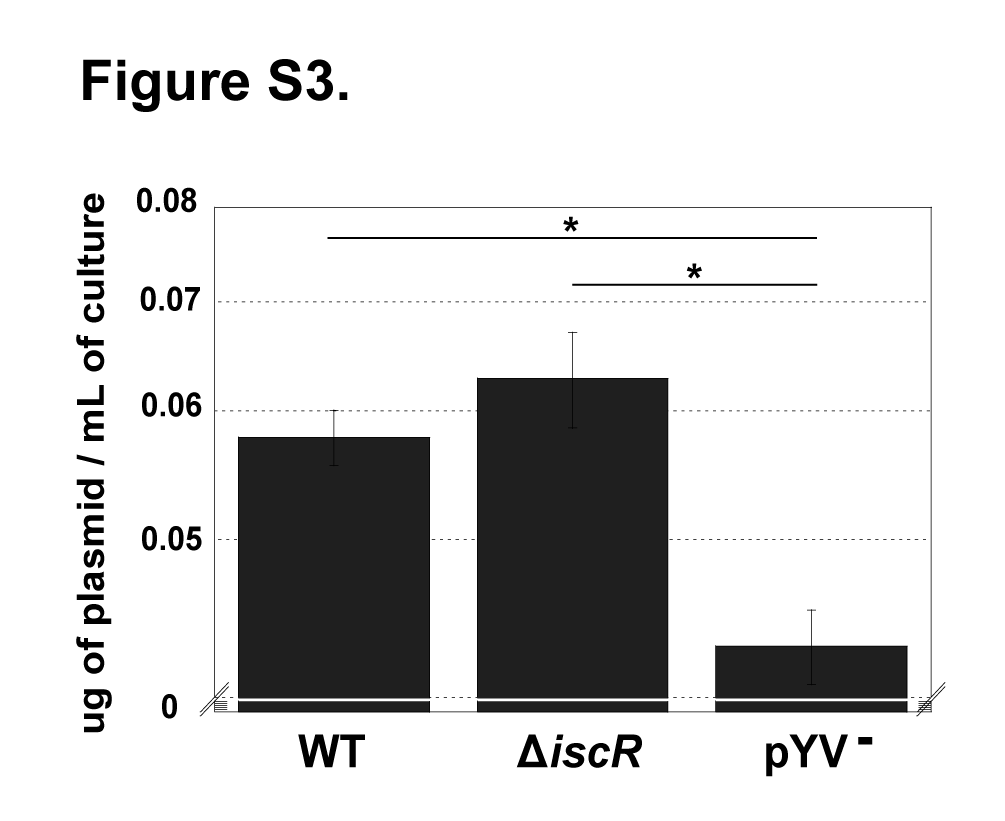

Supplement: Figure S3 — Mutation of iscR does not affect pYV virulence plasmid yield. Relative amounts of the virulence plasmid, pYV, were analyzed from standardized cultures of the wild type (WT), iscR mutant (ΔiscR) and pYV− strains grown in M9 at 37°C for 3 hours through midiprep analysis (Promega) according to the manufacturer's protocol. Plasmid yield was quantified via spectrophotometric analysis (Nanodrop). The data is displayed as µg of plasmid isolated per mL of culture ± SEM and is an average of 3 independent experiments. *p≤0.05, as determined by Student t test. (TIF) [file ppat.1004194.s003.tif]

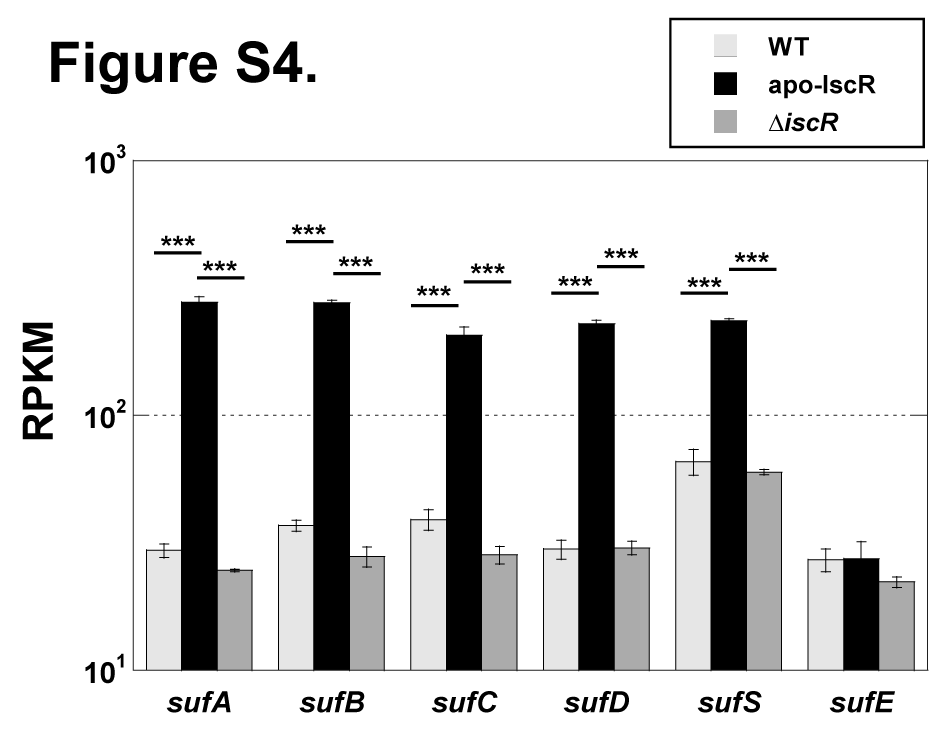

Supplement: Figure S4 — Expression of the suf operon is increased in the apo-locked IscR mutant strain. RNAseq analysis was performed on WT, ΔiscR and apo-IscR Y. pseudotuberculosis strains after growth in M9 at 37°C for 3 h (T3SS-inducing conditions). The data is presented as mean RPKM ± SEM and is an average of 3 independent experiments. ***p≤0.0001, as determined by Bayseq followed by FDR post hoc test. (TIF) [file ppat.1004194.s004.tif]

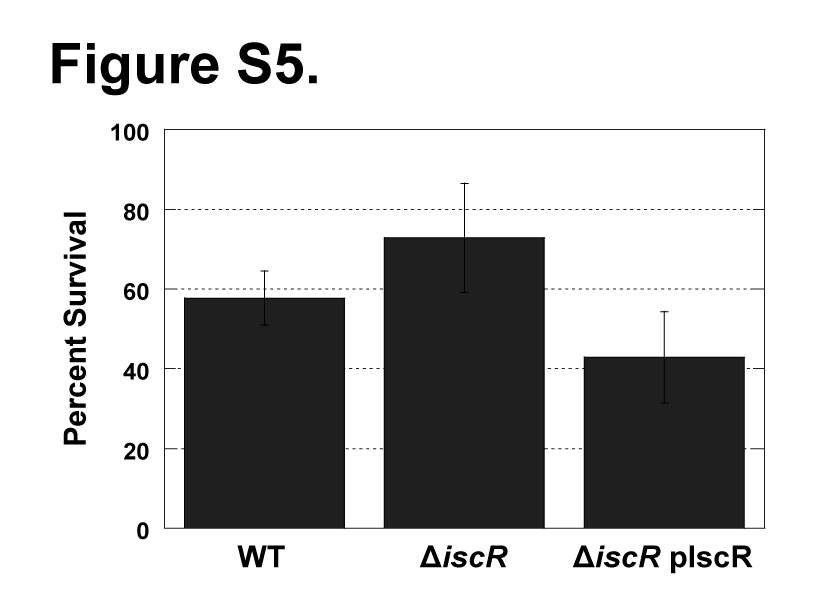

Supplement: Figure S5 — IscR is not required for survival post-exposure to hydrogen peroxide stress. Hydrogen peroxide assays were performed similar to Schiano et al. [87]. Y. pseudotuberculosis wild type (WT), ΔiscR, and iscR complemented (ΔiscR pIscR) strains were grown overnight in 2xYT at 26°C. Cultures were standardized to an OD600 of 0.1 and grown at 26°C with shaking to mid-log phase, at which point they were diluted 1∶10 into fresh 2xYT. Samples were supplemented with 50 µl of either sterile water (negative control) or hydrogen peroxide to a final concentration of 50 mM. Samples were incubated with shaking at 26°C and CFU determined via serial dilution and plating 10 min after the start of treatment. The data is displayed as percent survival (CFU H2O2/CFU H2O)*100) ± SEM and is an average of 3 independent experiments. (TIF) [file ppat.1004194.s005.tif]
