# Supplementary material for: IscR Is Essential for Yersinia pseudotuberculosis Type III Secretion and Virulence
Source: PLoS Pathog. 2014 Jun 12;10(6):e1004194. doi: 10.1371/journal.ppat.1004194 (PMC4055776; doi:10.1371/journal.ppat.1004194)
Supplement: Table S2 — Total pYV-encoded genes differentially regulated by IscR, identified by RNAseq analysis. (DOCX) [file ppat.1004194.s007.docx]

**Table S2. pYV Genes Differentially Regulated by IscR, Identified by RNAseq Analysis.**

| **Gene ID** | **Gene product/function** | **Gene Name** | **Operon Structure** | **Fold DOWN**  **Apo-IscR** | **Fold DOWN**  **Δ*iscR*** | **LcrF/VirF Regulated^a^** |
| --- | --- | --- | --- | --- | --- | --- |
| pYV0001 | serine/threonine kinase effector protein | *ypkA/yopO* |  |  |  |  |
| pYV0002 | hypothetical protein |  |  | -2.1 | -4.6 |  |
| pYV0003 | transposase remnant |  |  |  | -2.2 |  |
| pYV0004 | hypothetical protein |  |  |  |  |  |
| pYV0005 | replication initiation protein | *repA* | ***repBA*** |  |  |  |
| pYV0006 | hypothetical protein |  |  |  |  |  |
| pYV0007 | replication transcriptional regulator | *copB* |  |  |  |  |
| pYV0008 | transposase remnant |  |  | -2.6 | -2.6 |  |
| pYV0009 | hyothetical protein |  |  | -2.6 | -3.3 |  |
| pYV0010 | hypothetical protein |  |  | -4.0 | -3.3 |  |
| pYV0011 | transposase remname |  |  |  |  |  |
| pYV0012 | hypothetical protein |  |  | -5.2 | -4.2 |  |
| pYV0013 | *Yersinia* adhesin | *yadA* |  | -3.7 |  |  |
| pYV0014 | transposase remnant |  |  | -3.1 | -3.2 |  |
| pYV0015 | transposase remnant |  |  | -3.7 | -3.9 |  |
| pYV0016 | transposase protein | *tnpA* |  | -2.5 | -2.6 |  |
| pYV0017 | putative resolvase |  |  |  |  |  |
| pYV0018 | putative transposase |  |  |  |  |  |
| pYV0019 | putative transposase |  |  |  |  |  |
| pYV0020 | YopH targeting protein | *sycH* |  |  |  |  |
| pYV0021 | putative transposase |  |  | -2.3 | -2.4 |  |
| pYV0022 | putative transposase |  |  |  | -2.3 |  |
| pYV0023 | transposase remnant |  |  | -3.0 | -2.8 |  |
| pYV0024 | YopE chaperone | *sycE* |  |  |  | X |
| pYV0025 | outer membrane virulence protein | *yopE* |  | -2.7 |  | X |
| pYV0026 | hypothetical protein |  |  |  |  |  |
| pYV0027 | hypothetical protein |  |  |  |  |  |
| pYV0028 | transposase remnant |  |  |  |  |  |
| pYV0029 | hypothetical protein |  |  |  |  |  |
| pYV0030 | hypothetical protein |  |  |  |  |  |
| pYV0031 | plasmid partitioning control protein | *sopB* |  |  |  |  |
| pYV0032 | plasmid partitioning transcription repressor | *sopA* |  |  |  |  |
| pYV0033 | hypothetical protein |  |  |  |  |  |
| pYV0034 | transposase remnant |  |  |  | -2.3 |  |
| pYV0035 | transposase |  |  | -2.9 | -3.0 |  |
| pYV0036 | transposase |  |  | -3.0 | -3.6 |  |
| pYV0037 | pseudogene |  |  | -2.7 | -4.2 |  |
| pYV0038 | pseudogene |  |  | -4.6 | -8.3 |  |
| pYV0039 | putative transposase |  |  | -5.6 | -7.5 |  |
| pYV0040 | yop targeting protein | *yopK* |  | -7.1 | -9.3 | X |
| pYV0041 | yop targeted effector | *yopT* |  |  | -5.5 | X |
| pYV0042 | yopT chaperone |  |  |  |  |  |
| pYV0043 | transposase remnant |  |  |  |  |  |
| pYV0044 | hypothetical protein |  |  |  | -4.1 |  |
| pYV0045 | hypothetical protein |  |  |  |  |  |
| pYV0046 | transposase remnant |  |  |  | -2.9 |  |
| pYV0047 | effector protein | *yopM* |  | -3.0 | -5.3 | X |
| pYV0048 | hypothetical protein |  |  |  |  |  |
| pYV0049 | hypothetical protein |  |  |  | -2.4 |  |
| pYV0050 | hypothetical protein |  |  |  |  |  |
| pYV0051 | hypothetical protein |  |  |  |  |  |
| pYV0052 | hypothetical protein |  |  |  |  |  |
| pYV0053 | hypothetical protein |  |  |  |  |  |
| pYV0054 | Yop negative regulation/targeting component | *yopD* | ***lcrGVH-yopBD*** |  |  | X |
| pYV0055 | Yop targeting protein | *yopB* |  | -2.0 |  | X |
| pYV0056 | low calcium response protein | *lcrH* |  |  | -3.9 | X |
| pYV0057 | putative V antigen, antihost protein/regulator | *lcrV* |  |  | -3.5 | X |
| pYV0058 | Yop regulator | *lcrG* |  |  | -2.8 | X |
| pYV0059 |  | *lcrR* | ***virA*** |  |  |  |
| pYV0060 | membrane-bound Yop protein | *yscV* |  |  |  |  |
| pYV0061 | type III secretion protein | *yscY* |  |  | -2.2 |  |
| pYV0062 | type III secretion protein | *yscX* |  |  | -2.5 |  |
| pYV0063 | type III secretion protein | *sycN* |  |  | -2.5 |  |
| pYV0064 | Yop secretion and targeting protein | *tyeA* |  |  | -2.1 |  |
| pYV0065 | membrane-bound Yop targeting protein | *yopN* |  |  |  |  |
| pYV0066 | hypothetical protein |  |  |  |  |  |
| pYV0067 | ATP synthase | *yscN* | ***virB*** |  |  |  |
| pYV0068 | type III secretion protein | *yscO* |  |  | -2.0 |  |
| pYV0069 | type III secretion protein | *yscP* |  |  | -2.1 |  |
| pYV0070 | type III secretion protein | *yscQ* |  |  |  |  |
| pYV0071 | Yop secretion membrane protein | *yscR* |  |  |  |  |
| pYV0072 | type III secretion protein | *yscS* |  |  |  |  |
| pYV0073 | type III secretion protein | *yscT* |  |  |  |  |
| pYV0074 | type III secretion protein | *yscU* |  |  |  |  |
| pYV0075 | Yop targeting lipoprotein | *virG*  */yscW* | ***yscW-lcrF*** |  | -2.5 | X |
| pYV0076 | thermoregulatory protein | *lcrF* |  |  | -3.3 | X |
| pYV0077 | type III secretion protein | *yscA* | ***virC*** |  |  | X |
| pYV0078 | type III secretion protein | *yscB* |  |  | -3.5 | X |
| pYV0079 | type III secretion protein | *yscC* |  |  | -2.0 | X |
| pYV0080 | type III secretion protein | *yscD* |  |  | -2.0 | X |
| pYV0081 | type III secretion protein | *yscE* |  |  |  | X |
| pYV0082 | type III secretion protein | *yscF* |  |  | -2.8 | X |
| pYV0083 | type III secretion protein | *yscG* |  |  | -2.9 | X |
| pYV0084 | type III secretion protein | *yscH* |  |  | -2.1 | X |
| pYV0085 | type III secretion protein | *yscI* |  |  |  | X |
| pYV0086 | type III secretion lipoprotein | *yscJ* |  |  |  | X |
| pYV0087 | type III secretion protein | *yscK* |  |  | -3.2 | X |
| pYV0088 | type III secretion protein | *yscL* |  |  | -2.2 | X |
| pYV0089 | type III secretion regulatory protein | *lcrQ* |  |  | -2.1 | X |
| pYV0090 | putative transposase |  |  | -2.3 | -2.7 |  |
| pYV0091 | putative transposase |  |  | -2.6 | -3.1 |  |
| pYV0092 | putative transposase |  |  | -2.6 | -3.2 |  |
| pYV0093 | putative transposase |  |  |  | -2.2 |  |
| pYV0094 | protein-tyrosine phosphatase Yop effector | *yopH* |  | -4.4 |  | X |
| pYV0095 | putative transposase |  |  |  |  |  |
| pYV0096 | transposase, IS630 family |  |  |  |  |  |
| pYV0097 | hypothetical protein |  |  |  |  |  |
| pYV0098 | effector protein | *yopJ* |  |  | -3.4 | X |
| pYV0099 | hypothetical protein |  |  |  | -4.8 |  |

^a^Genes indicated as being regulated by LcrF/VirF are from previously published reports [18-24].
