# Supplementary material for: IscR Is Essential for Yersinia pseudotuberculosis Type III Secretion and Virulence
Source: PLoS Pathog. 2014 Jun 12;10(6):e1004194. doi: 10.1371/journal.ppat.1004194 (PMC4055776; doi:10.1371/journal.ppat.1004194)
Supplement: Table S3 — Y. pseudotuberculosis primers used in this study. (DOCX) [file ppat.1004194.s008.docx]

| **Table S3. *Y. pseudotuberculosis* primers used in this study.** | | |
| --- | --- | --- |
| **Name** | **Primer Sequence^a^** | **References** |
| R3’*iscR* | CAGTTTACTCAGATCAACAGG | This work |
| F35*’iscR* | TTTAGCTATGACGATTAACGTCAATCTGC | This work |
| R5*’iscR* | CGTTAATCGTCATAGCTAAAGTTACCTGT | This work |
| F5*’iscR* | CAGTGATGTTATTGGTAAGGCG | This work |
| F5’ apo-IscR | gatacggcgggtaagagtaa | This work |
| R5’ apo-IscR | gccctgagcgccttctttaccctgagcacgggttgcatcaa | This work |
| F3’ apo-IscR | agaaggcgctcagggcggaaatcgcgccctgacacataccc | This work |
| R3’ apo-IscR | caaacttatgcgaacgtgag | This work |
| Fi*scR*C | ATGATGGGATCCGCCAATCCTGAATATAGCTCC | This work |
| R*iscR*C | ATGATGGTCGACTATGCTCCGTACATCACTTCC | This work |
| Fq*iscS* | CGACGCCAGTAGATCCGCGT | This work |
| Rq*iscS* | ACGAGGGTCTGCACCCACCA | This work |
| Fq*erpA* | TACCGGTGGTGGATGTAGCGGG | This work |
| Rq*erpA* | ATAATCCACGGCACCGCCCAC | This work |
| Fq*yscN* | CTTCGCTTATTCGTAGTGCT | This work |
| Rq*yscN* | TCGCCTAAATCAGACTCAAT | This work |
| Fq*yscF* | TCTCTGGATTTACGAAAGGA | This work |
| Rq*yscF* | GCTTATCTTTCAATGCTGCT | This work |
| Fq*lcrF* | GGAGTGATTTTCCGTCAGTA | This work |
| Rq*lcrF* | CTCCATAAATTTTTGCAACC | This work |
| Fq16s | AGCCAGCGGACCACATAAAG | [[72](#_ENREF_72)] |
| Rq16s | AGTTGCAGACTCCAATCCGG | [[72](#_ENREF_72)] |
| *hya* top | ATAAATCCACACAGTTTGTATTGTTTTGTG | Nesbit 2006 |
| *hya* bottom | CACAAAACAATACAAACTGTGTGGATTTAT | Nesbit 2006 |
| *lcrF* top | TTTAAAACCATATATACAGTATGGTAATTGTATT | This work |
| *lcrF* bottom | AATACAATTACCATACTGTATATATGGTTTTAAA | This work |
| *mlcrF* top | TTGAAAAGTATCTATACAGGCTAATAATTGCATT | This work |
| *mlcrF* bottom | AATGCAATTATTAGCCTGTATAGATACTTTTCAA | This work |
| *iscR* top | AAATAGTTGACTAAAACACTCAAGAATGTC | This work |
| *iscR* bottom | GACATTCTTGAGTGTTTTAGTCAACTATTT | This work |

^a^ Restriction sites, if any, are underlined
