# Supplementary material for: IscR Is Essential for Yersinia pseudotuberculosis Type III Secretion and Virulence
Source: PLoS Pathog. 2014 Jun 12;10(6):e1004194. doi: 10.1371/journal.ppat.1004194 (PMC4055776; doi:10.1371/journal.ppat.1004194)
Supplement: Table S4 — Known type 2 DNA-binding sequences used for in silico search. (DOCX) [file ppat.1004194.s009.docx]

| **Table S4. Known type II DNA-binding sequences used for *in silico* search.** | | | |
| --- | --- | --- | --- |
| **Gene ID** | **IscR Site** | **References** | |
| *sufA* | GGtAaAgCCCCTgCGTTTGCtGGGTTgAA | | [31] |
| *ydiU* | GCGATAACCCtTCtGTTTGCtGGtgTTtA | | [31] |
| *malM* | GCGAaAgCCCCTCtGaTTatcGGGTTTAg | | [31] |
| *hyaA* | GatAaAtCCaCaCAGTTTGtAttGTTTtg | | [31] |
| *b4140* | tCGcaAcCCatTCAGgTaGCcGGGgTTAA | | [31] |
| *yahC* | cCGATAcCCttTCAcTcaatcaGGTTTtA | | [31] |
